# High risk of non-cancer mortality in bladder cancer patients: evidence from SEER-Medicaid

**DOI:** 10.1007/s00432-023-04867-z

**Published:** 2023-06-03

**Authors:** Shunde Wang, Chengguo Ge

**Affiliations:** 1 Department of Urology, The ChenJiaqiao Hospital of ShaPingba District of Chongqing City, Chongqing, 401331 People’s Republic of China; 2 Department of Urology, The Second Affiliated Hospital of Chongqing Medical University, No.76, Linjiang Road, Yuzhong District, Chongqing, 400010 People’s Republic of China

**Keywords:** Bladder cancer, Standardized mortality ratio, Non-cancer cause of death, Competing risk analysis, SEER

## Abstract

**Purpose:**

The objective of this study was to investigate non-cancer causes of death and associated risk factors after bladder cancer (BC) diagnosis.

**Methods:**

Eligible BC patients were obtained from the SEER database. SEER*Stat software 8.3.9.2 was used to calculate the standardized mortality ratios (SMRs). The proportions of different non-cancer cause of death were calculated and analyzed in different follow-up periods. Multivariate competing risk model was used to analyze the risk factors for death of BC and non-cancer diseases.

**Results:**

In total, 240,954 BC patients were included and 106,092 patients experienced death, with 37,205 (35.07%), 13,208 (12.45%) and 55,679 (52.48%) patients experienced BC, other cancer and non-cancer disease-related deaths, respectively. Overall SMR for BC patients who died from non-cancer diseases was 2.42 (95% CI [2.40–2.44]). Cardiovascular diseases were the most common non-cancer cause of death, followed by respiratory diseases, diabetes mellitus, and infectious diseases. Multivariate competing risk analysis identified the following high-risk factors for non-cancer mortality: age > 60 years, male, whites, in situ stage, pathological type of transitional cell carcinoma, not receiving treatment (including surgery, chemotherapy, or radiation), and widowed.

**Conclusions:**

Cardiovascular diseases are the leading non-cancer cause of death in BC patients, followed by respiratory disease, diabetes mellitus and infectious diseases. Physicians should pay attention to the risk of death from these non-cancer diseases. Also, physicians should encourage patients to engage in more proactive self-surveillance and follow up.

**Supplementary Information:**

The online version contains supplementary material available at 10.1007/s00432-023-04867-z.

## Introduction

Bladder cancer (BC) is estimated to account for more than 500,000 new cases and 200,000 deaths per year worldwide. In the United States alone, there are more than 80,000 new cases and 17,000 deaths each year (Richters et al. 2020; Lenis et al. 2020; Siegel et al. 2019, 2020).

With the updating of various anti-cancer treatment strategies, cancer-related mortality has gradually decreased (Kochanek et al. 2019a; Robertson et al. 2018; Jalanko et al. 2020). As a result, non-cancer causes of death are of increasing concern as survival times increase. Studies have shown that non-cancer diseases such as heart disease are the leading cause of non-cancer mortality in cancer patients. In most cancer patients, the mortality rate from non-cancer diseases even exceeds that from primary cancer (Siegel et al. 2021; Abdel-Rahman 2017; Zhang et al. 2021; Sturgeon et al. 2019).

With the increasing quest for longer survival and higher quality of life, deaths due to non-cancer diseases should be taken into account. Studying non-cancer causes of death allows us to more accurately evaluate the risk of death in patients with cancer. As a result, we can provide early intervention and management. The focus of this study was on standardized mortality ratios (SMRs) for non-cancer causes of death, and on high-risk factors that can contributed to experiencing non-cancer mortality in BC patients.

## Materials and methods

### Data source

Patients information was obtained from the Surveillance, Epidemiology, and End Results (SEER) database, which includes approximately 28% of the general US population, and used the SEER*Stat software 8.3.9.2 to access the database: Incidence—SEER Research Plus Data, 18 Registries (excl AK), Nov 2020 Sub (2000–2018) for SMRs.

### Patients

We included patients who were pathologically diagnosed with BC between 2000 and 2017. Cases diagnosed by death certificate and autopsy were excluded. Cases with incomplete information were also excluded.

### Standardized mortality ratio (SMR)

For patients with BC in the SEER database, we calculated the number of deaths under different variables. The focus of this study was on non-cancer mortality in patients with BC. We classify non-cancer diseases into the following six categories: infectious diseases, diabetes mellitus, cardiovascular diseases, respiratory diseases, digestive diseases, and other non-cancer diseases. Supplementary Table 1 shows the definitions of non-cancer causes of death and the ICD-10 codes for the diseases.

First, the SMRs were calculated for all causes of deaths (BC, other cancers, non-cancer diseases). We then calculated SMRs for non-cancer causes of death for BC patients at different follow-up period (< 1 year, 1–5 years, and > 5 years) after diagnosis, with the distribution of the population and the age (mean ± SD) by variables. SMR was defined as the observed-to-expected ratio; the observation population was defined as patients diagnosed with BC in the United States from 2000 to 2017, which was collected from the SEER database. The competing risk analysis using Fine-Gray model was performed to adjust for confounding effects of age, sex, race, summary stage, year of diagnosis, histologic type, treatment (surgery, chemotherapy, and radiation therapy), and marital status to evaluate risks for non-cancer diseases mortality and BC mortality, and to plot cumulative mortality curves.

### Statistical analysis

All analyses were performed using SEER*Stat software (version 8.3.9.2), R 4.1.1 (R foundation for Statistical Computing, Vienna, Austria), and Microsoft Excel 2019 (Microsoft, Redmond, WA). All statistical analyses were two-sided with a *p* value < 0.05 being considered statistically significant.

## Results

### Baseline characteristics

**Fig. 1:**
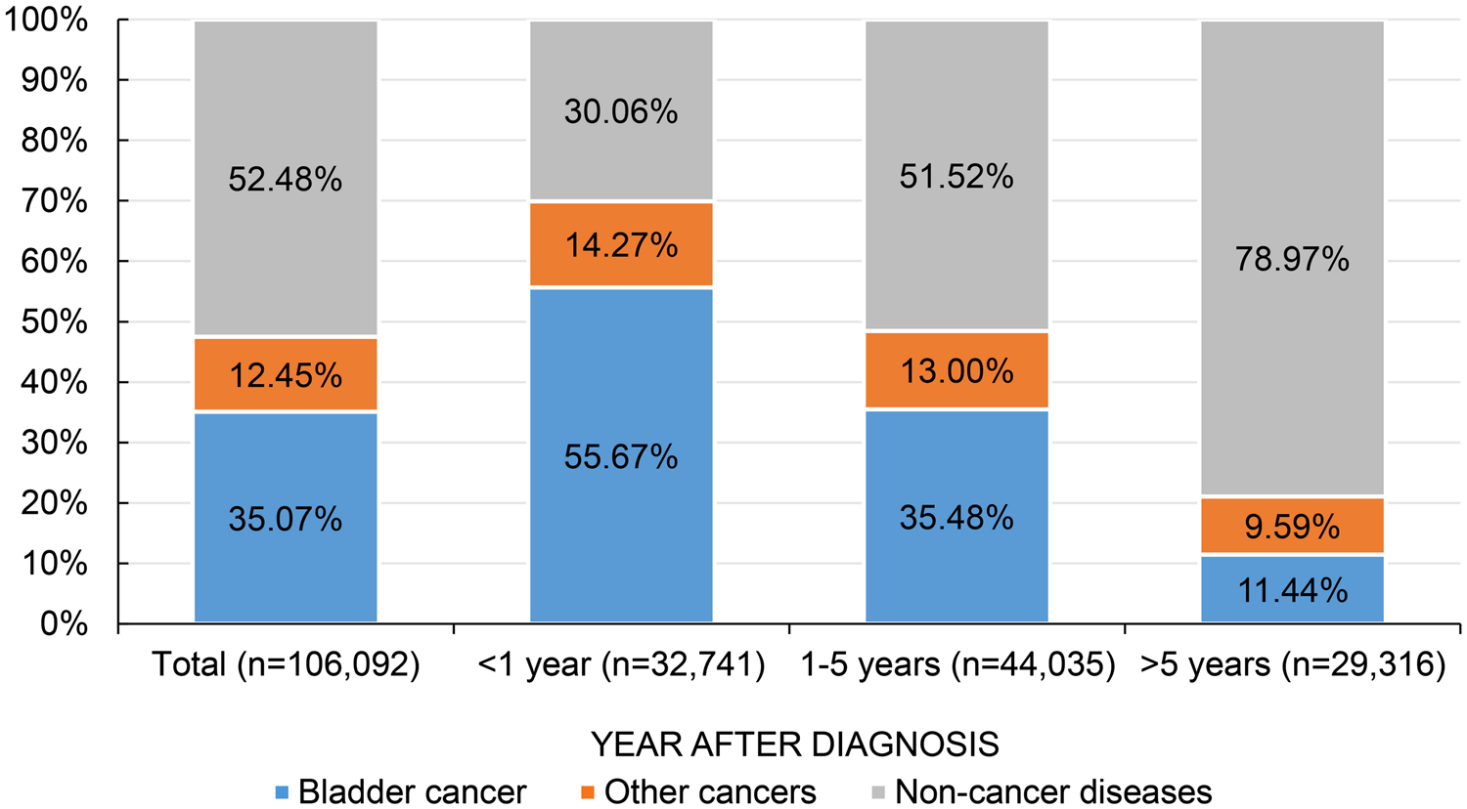
Percentage of all causes of death in bladder cancer patients at different follow-up latency

Cumulatively, 240,954 eligible BC patients were obtained from the SEER database, of which 106,092 experienced mortality. Among all the causes of death in BC patients, only the proportion of non-cancer diseases increased with increasing follow-up time (Fig. 1), from 30.06 to 78.97%. Supplementary Table 2 shows the SMR for all causes of death in BC patients after diagnosis, the overall SMR was 3.23 (95% CI [3.21–3.25]), with BC has the highest SMR (SMR [95% CI] 7.59 [7.52–7.67]), followed by other cancers (SMR [95% CI] 2.68 [2.63–2.72]), and non-cancer diseases (SMR [95% CI] 2.42 [2.40–2.44]). Of all death in BC patients, 55,679 patients died from non-cancer diseases with a mean age at death of 75.84 ± 8.71 years. 52,803 (94.83%) patients were older than 60 years at diagnosis, 42,901 (77.05%) were male patients and 51,269 (92.08%) were white. The highest proportion of deaths was observed during the > 5 years follow-up period (41.58%), followed by 1–5 years (40.74%) after diagnosis. Table 1 shows the baseline characteristics of patients with BC who experienced non-cancer mortality: the number of deaths at different follow-up periods (< 1 year, 1–5 years, and > 5 years), and the mean ± SD age at diagnosis. Figure 1 shows the proportion of each cause of death in BC patients at different follow-up period.

**Table 1:** Baseline characteristics of bladder cancer patients under non-cancer causes of death

Factors	Overall		< 1 year		1–5 years		> 5 years	
	No.	Age (mean ± SD)	No.	Age (mean ± SD)	No.	Age (mean ± SD)	No.	Age (mean ± SD)
Total	55,679	75.84 ± 8.71	9843	77.67 ± 8.62	22,686	77.41 ± 8.33	23,150	74.09 ± 8.68
Age (years)								
00–60	2876	54.64 ± 5.42	421	54.14 ± 6.13	945	54.70 ± 5.12	1510	54.70 ± 5.43
60+	52,803	77.29 ± 6.78	9422	78.96 ± 6.66	21,741	78.59 ± 6.58	21,640	75.76 ± 6.64
Sex								
Male	42,901	75.57 ± 8.69	7521	77.49 ± 8.59	17,948	77.16 ± 8.33	17,432	73.75 ± 8.63
Female	12,778	76.80 ± 8.71	2322	78.26 ± 8.70	4738	78.35 ± 8.25	5718	75.27 ± 8.76
Race								
White	51,269	75.97 ± 8.59	8873	77.87 ± 8.41	20,914	77.59 ± 8.19	21,482	74.19 ± 8.58
Black	2673	72.63 ± 10.49	621	74.58 ± 10.87	1104	73.74 ± 10.16	948	70.81 ± 10.34
Other races^a^	1737	76.97 ± 8.18	349	78.16 ± 8.52	668	77.89 ± 7.90	720	75.86 ± 8.14
Summary stage								
In situ	30,755	76.07 ± 8.66	3586	78.50 ± 8.02	12,603	77.66 ± 8.24	14,566	74.44 ± 8.76
Localized	21,685	76.06 ± 8.49	4745	78.41 ± 8.03	9089	77.63 ± 8.11	7851	73.95 ± 8.46
Regional	2530	73.20 ± 9.42	1026	74.49 ± 9.73	826	73.78 ± 9.53	678	71.93 ± 8.96
Distant	709	72.39 ± 10.54	486	72.55 ± 11.33	168	73.09 ± 9.87	55	71.21 ± 9.64
Year of diagnosis								
2000–2005	26,325	75.01 ± 8.67	3370	77.87 ± 8.32	8396	77.29 ± 8.09	14,559	73.50 ± 8.63
2006–2011	19,620	76.45 ± 8.64	3212	77.60 ± 8.70	8382	77.56 ± 8.38	8026	75.15 ± 8.68
2012–2017	9734	77.36 ± 8.66	3261	77.53 ± 8.88	5908	77.37 ± 8.61	565	76.52 ± 8.14
Histologic type								
Tcc	53,672	76.08 ± 8.69	9144	77.86 ± 8.40	21,994	77.52 ± 8.27	22,534	74.31 ± 8.80
Scc	804	73.69 ± 9.83	272	75.39 ± 10.63	247	75.59 ± 9.63	285	71.63 ± 9.16
Nec	213	73.63 ± 7.92	101	75.18 ± 11.32	71	75.39 ± 9.02	41	73.06 ± 7.25
Ac	362	74.40 ± 9.03	110	74.51 ± 9.73	149	75.25 ± 7.74	103	73.49 ± 9.41
Oet	628	74.93 ± 8.91	216	77.03 ± 9.76	225	77.49 ± 8.20	187	73.34 ± 8.68
Surgery								
No	2710	75.15 ± 8.45	753	77.15 ± 9.94	1043	77.25 ± 8.18	914	73.55 ± 7.81
TURBT	49,655	76.32 ± 8.58	8194	78.42 ± 8.06	20,578	77.79 ± 8.11	20,883	74.46 ± 8.72
PC	599	74.90 ± 8.17	142	77.18 ± 9.21	211	76.52 ± 8.40	246	74.03 ± 7.83
RC	2715	71.09 ± 9.38	754	71.66 ± 9.61	854	71.37 ± 9.67	1107	70.73 ± 9.12
Radiation therapy								
Yes	2345	75.06 ± 8.33	831	77.37 ± 8.68	1062	76.84 ± 8.40	452	73.04 ± 7.65
No/unknown	53,334	75.89 ± 8.73	9012	77.70 ± 8.62	21,624	77.44 ± 8.32	22,698	74.17 ± 8.74
Chemotherapy								
Yes	6702	74.56 ± 9.37	1435	74.69 ± 9.66	3143	75.66 ± 9.13	2124	73.18 ± 9.35
No/unknown	48,977	76.01 ± 8.60	8408	78.18 ± 8.33	19,543	77.69 ± 8.16	21,026	74.19 ± 8.60
Marital status								
Married	32,992	75.44 ± 8.29	5163	77.51 ± 8.11	13,057	77.13 ± 7.98	14,772	73.82 ± 8.20
Separated	323	71.62 ± 9.92	47	72.09 ± 9.00	144	72.77 ± 10.11	132	70.39 ± 9.90
Divorced	3924	71.07 ± 9.52	733	72.42 ± 9.34	1619	72.45 ± 9.32	1572	69.53 ± 9.50
Widowed	13,497	80.24 ± 5.96	2866	81.60 ± 5.20	58,85	81.17 ± 5.45	4746	78.65 ± 6.43
Unmarried	4943	71.32 ± 11.16	1034	71.81 ± 11.77	19,81	72.69 ± 10.91	1928	69.96 ± 11.00

*SMR* standardized mortality ratio, *CI* confidence interval, *Tcc* transitional cell carcinoma, *Scc* squamous cell carcinoma, *Nec* neuroendocrine carcinoma, *Ac* adenocarcinoma, *Oet* other epithelial tumors, *TURBT* transurethral resection of bladder tumor, *PC* partial cystectomy, *RC* radical cystectomy

^a^Including American Indian/Alaska Native and Asian or Pacific Islander

### SMR after BC diagnosis

**Fig. 2:**
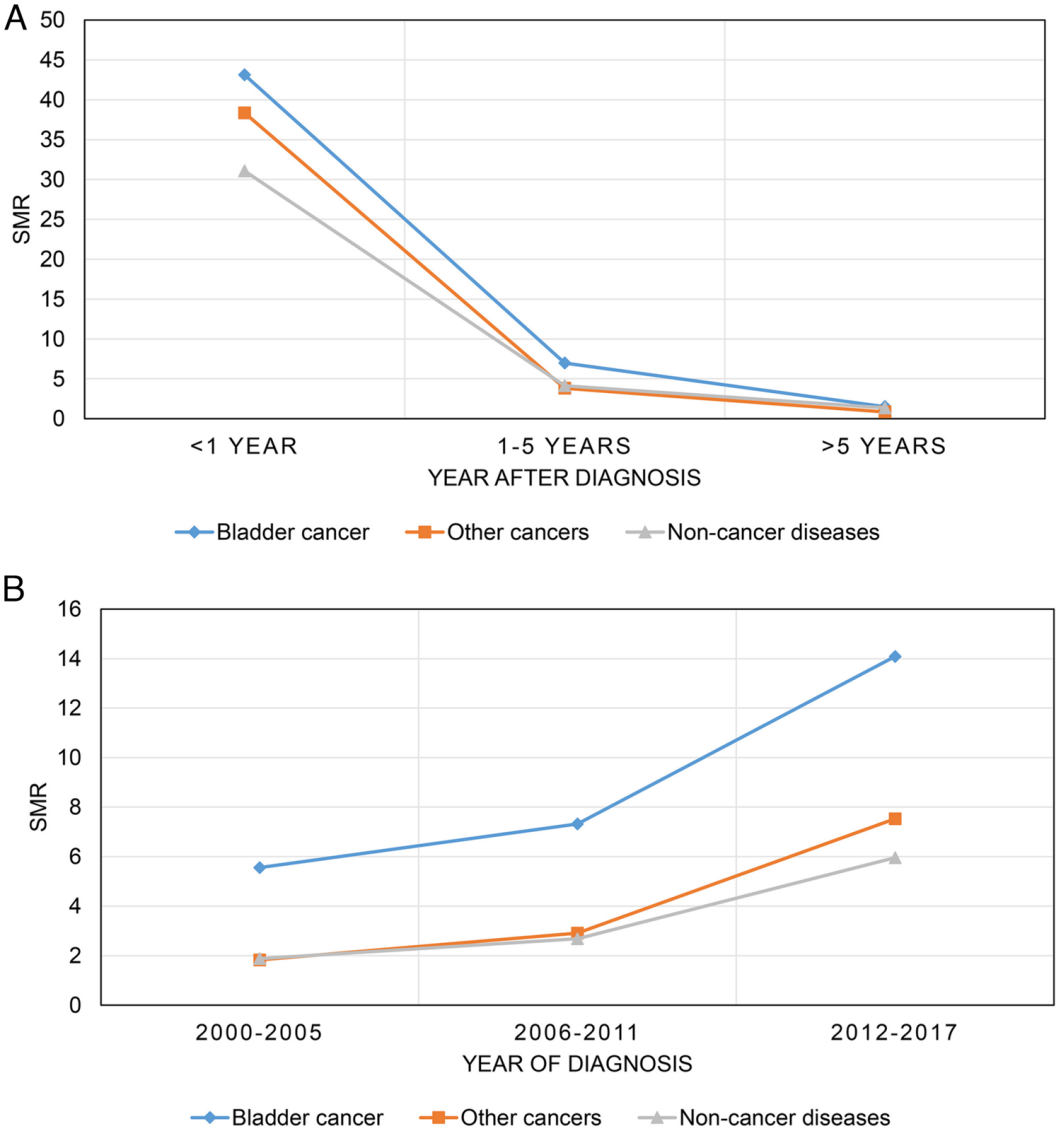
Trends in standardized mortality ratios for all causes of death in patients with bladder cancer at different follow-up latency (**A**) and different year of diagnosis (**B**)

Figure 2A shows that SMR decreases with increasing follow-up time. At < 1 year follow-up period, the highest SMR was for BC, followed by other cancers, and then non-cancer diseases, while at 1–5 years follow-up period, the SMR for non-cancer diseases surpassed other cancers in second place. Figure 2B shows that SMR increases with increasing year of diagnosis, while BC consistently has the highest level of SMR.

**Fig. 3:**
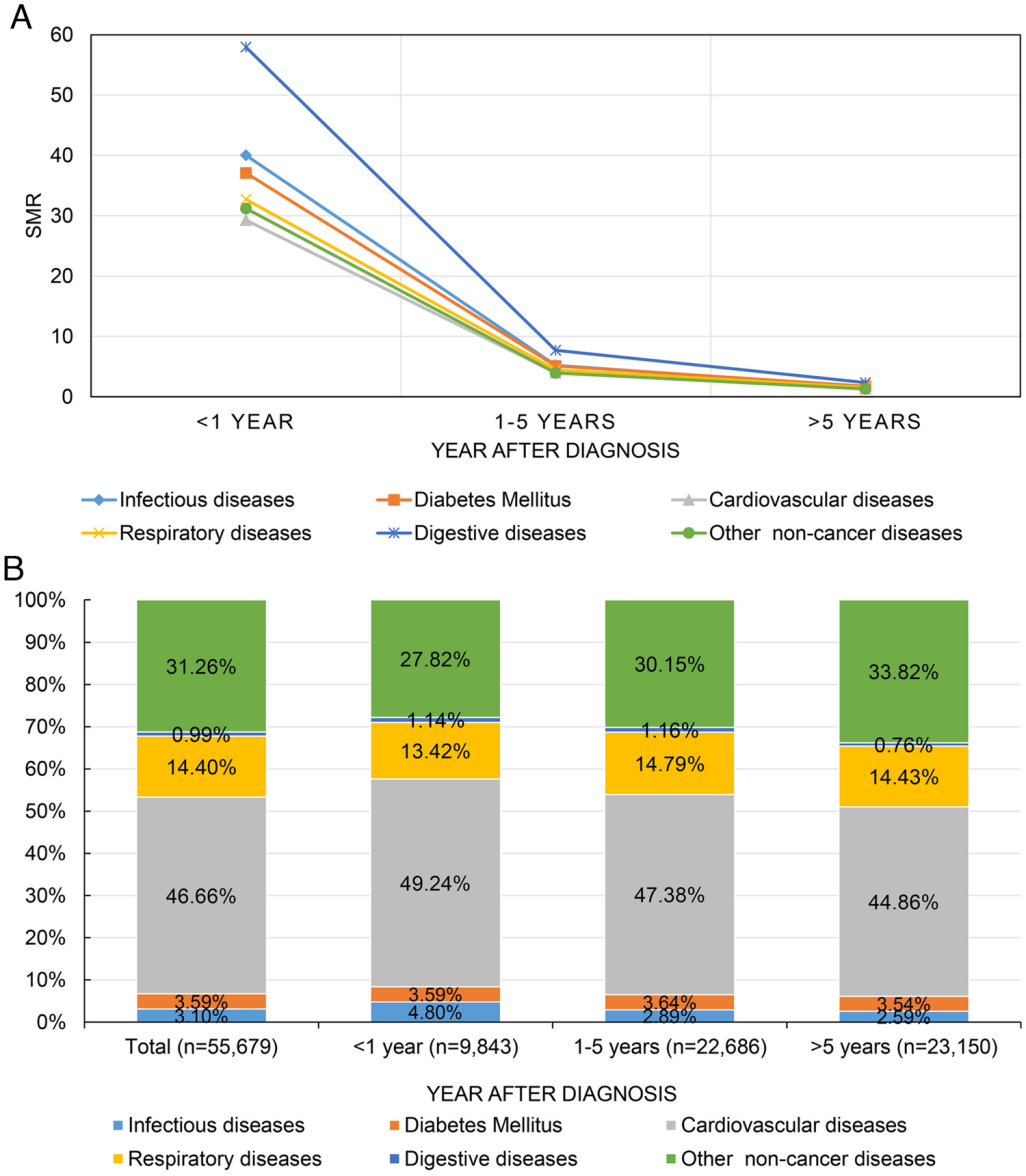
Trends in standardized mortality ratios (**A**) and proportions (**B**) of all non-cancer causes of death for patients with bladder cancer at different follow-up period after diagnosis

Figure 3A shows that the SMR for the six non-cancer diseases decreased with increasing follow-up time, with digestive diseases consistently having the highest SMR, followed by infectious diseases and diabetes mellitus, while cardiovascular diseases consistently having the lowest SMR. Figure 3B shows the proportion of the six non-cancer diseases at different follow-up periods, with cardiovascular diseases consistently having the highest proportion, followed by other non-cancer diseases and respiratory diseases.

Table 2 shows the SMR for non–cancer cause of death in BC patients after diagnosis, the overall SMR was 2.42 (95% CI [2.40–2.44]). SMR was always the highest within one year after diagnosis. Table 3 shows the SMR for all non–cancer causes of death in BC patients after diagnosis, the highest SMR was observed in digestive diseases (SMR [95% CI] 4.95 [4.55–5.38]), followed by infectious diseases (SMR [95% CI] 3.33 [3.17–3.49]) and diabetes mellitus (SMR [95% CI] 3.11 [2.98–3.25]).

**Table 2:** SMR for non–cancer cause of death in bladder cancer patients after diagnosis

Factors	Overall		< 1 year		1–5 years		> 5 years	
	Observed	SMR [95% CI]	Observed	SMR [95% CI]	Observed	SMR [95% CI]	Observed	SMR [95% CI]
Total	55,679	2.42* [2.40–2.44]	9843	31.07* [30.46–31.69]	22,686	4.12* [4.07–4.18]	23,150	1.35* [1.33–1.37]
Age (years)								
00–60	2876	13.71* [13.22–14.22]	421	351.91* [319.09–387.18]	945	40.62* [38.08–43.30]	1510	8.15* [7.74–8.57]
60+	52,803	2.32* [2.30–2.34]	9422	29.85* [29.25–30.46]	21,741	3.97* [3.91–4.02]	21,640	1.27* [1.26–1.29]
Sex								
Male	42,901	2.41* [2.38–2.43]	7521	30.07* [29.39–30.76]	17,948	4.04* [3.98–4.10]	17,432	1.33* [1.31–1.35]
Female	12,778	2.48* [2.44–2.52]	2322	34.81* [33.41–36.25]	4738	4.45* [4.33–4.58]	5718	1.42* [1.39–1.46]
Race								
White	51,269	2.37* [2.35–2.39]	8873	30.18* [29.56–30.82]	20,914	4.03* [3.98–4.08]	21,482	1.33* [1.31–1.35]
Black	2673	3.22* [3.10–3.34]	621	38.49* [35.52–41.64]	1104	5.30* [5.00–5.63]	948	1.56* [1.46–1.67]
Other races^a^	1737	3.40* [3.24–3.57]	349	52.07* [46.75–57.83]	668	6.28* [5.82–6.78]	720	1.81* [1.68–1.95]
Summary stage								
In situ	30,755	2.14* [2.12–2.17]	3586	27.11* [26.23–28.02]	12,603	3.93* [3.86–4.00]	14,566	1.32* [1.30–1.34]
Localized	21,685	2.71* [2.68–2.75]	4745	30.34* [29.48–31.21]	9089	4.22* [4.13–4.31]	7851	1.38* [1.35–1.41]
Regional	2530	4.40* [4.23–4.57]	1026	48.43* [45.51–51.49]	826	6.42* [5.99–6.87]	678	1.59* [1.48–1.72]
Distant	709	11.52* [10.69–12.40]	486	69.54* [63.50–76.01]	168	9.41* [8.04–10.94]	55	1.50* [1.13–1.95]
Year of diagnosis								
2000–2005	26,325	1.88* [1.85–1.90]	3370	29.82* [28.82–30.84]	8396	3.93* [3.85–4.01]	14,559	1.24* [1.22–1.26]
2006–2011	19,620	2.68* [2.65–2.72]	3212	31.71* [30.62–32.82]	8382	3.99* [3.90–4.07]	8026	1.57* [1.54–1.61]
2012–2017	9734	5.96* [5.84–6.07]	3261	31.81* [30.73–32.92]	5908	4.67* [4.55–4.79]	565	2.11* [1.94–2.29]
Histologic type								
Tcc	53,672	2.40* [2.38–2.42]	9144	30.36* [29.74–30.99]	21,994	4.10* [4.05–4.16]	22,534	1.35* [1.33–1.36]
Scc	804	3.11* [2.90–3.33]	272	47.98* [42.44–54.03]	247	4.63* [4.07–5.24]	285	1.43* [1.27–1.60]
Nec	213	4.68* [4.06–5.37]	101	53.45* [43.29–65.27]	71	5.91* [4.57–7.52]	41	1.35* [0.97–1.83]
Ac	362	3.66* [3.29–4.06]	110	39.05* [31.94–47.27]	149	6.14* [5.18–7.23]	103	1.47* [1.20–1.78]
Oet	628	3.31* [3.05–3.58]	216	40.49* [35.21–46.33]	225	4.03* [3.52–4.59]	187	1.44* [1.23–1.66]
Surgery								
No	2710	2.89* [2.78–3.00]	753	35.45* [32.97–38.08]	1043	4.14* [3.89–4.39]	914	1.38* [1.29–1.47]
TURBT	49,655	2.36* [2.34–2.38]	8194	29.38* [28.74–30.02]	20,578	4.04* [3.98–4.09]	20,883	1.33* [1.32–1.35]
PC	599	2.54* [2.34–2.76]	142	30.58* [25.75–36.04]	211	4.61* [4.01–5.28]	246	1.33* [1.17–1.51]
RC	2715	3.52* [3.39–3.66]	754	62.80* [58.39–67.44]	854	7.99* [7.46–8.54]	1107	1.70* [1.60–1.80]
Radiation therapy								
Yes	2345	4.10* [3.93–4.27]	831	31.66* [29.55–33.89]	1062	4.88* [4.59–5.18]	452	1.38* [1.25–1.51]
No/unknown	53,334	2.38* [2.36–2.40]	9012	31.01* [30.38–31.66]	21,624	4.09* [4.04–4.15]	22,698	1.35* [1.33–1.37]
Chemotherapy								
Yes	6702	3.30* [3.22–3.38]	1435	35.15* [33.36–37.02]	3143	4.74* [4.58–4.91]	2124	1.60* [1.54–1.67]
No/unknown	48,977	2.34* [2.32–2.36]	8408	30.46* [29.81–31.12]	19,543	4.04* [3.98–4.09]	21,026	1.33* [1.31–1.35]
Marital status								
Married	32,992	2.29* [2.27–2.32]	5163	31.77* [30.91–32.65]	13,057	4.17* [4.09–4.24]	14,772	1.33* [1.31–1.35]
Separated	323	3.33* [2.98–3.71]	47	57.94* [42.57–77.05]	144	6.02* [5.08–7.09]	132	1.83* [1.53–2.17]
Divorced	3924	3.66* [3.55–3.78]	733	51.92* [48.22–55.81]	1619	6.40* [6.10–6.72]	1572	1.96* [1.86–2.05]
Widowed	13,497	2.26* [2.22–2.30]	2866	24.70* [23.80–25.62]	5885	3.38* [3.29–3.47]	4746	1.15* [1.12–1.19]
Unmarried	4943	3.39* [3.29–3.48]	1034	44.32* [41.66–47.11]	1981	5.64* [5.39–5.89]	1928	1.78* [1.70–1.86]

*SMR* standardized mortality ratio, *CI* confidence interval, *Tcc* transitional cell carcinoma, *Scc* squamous cell carcinoma, *Nec* neuroendocrine carcinoma, *Ac* adenocarcinoma, *Oet* other epithelial tumors, *TURBT* transurethral resection of bladder tumor, *PC* partial cystectomy, *RC* radical cystectomy

^*^p < 0.05

^a^Including American Indian/Alaska Native and Asian or Pacific Islander

**Table 3:** SMR for all non–cancer causes of death in bladder cancer patients after diagnosis

Non-cancer diseases^a^	Overall		< 1 year		1–5 years		> 5 years	
	Observed	SMR [95% CI]	Observed	SMR [95% CI]	Observed	SMR [95% CI]	Observed	SMR [95% CI]
Total	55,679	2.42* [2.40–2.44]	9843	31.07* [30.46–31.69]	22,686	4.12* [4.07–4.18]	23,150	1.35* [1.33–1.37]
Infectious diseases	1728	3.33* [3.17–3.49]	472	40.03* [36.50–43.81]	656	5.21* [4.82–5.63]	600	1.57* [1.45–1.70]
Diabetes mellitus	1997	3.11* [2.98–3.25]	353	37.10* [33.33–41.18]	825	5.11* [4.76–5.47]	819	1.74* [1.62–1.86]
Cardiovascular diseases	25,981	2.39* [2.36–2.42]	4847	29.28* [28.47–30.12]	10,748	3.98* [3.91–4.06]	10,386	1.30* [1.27–1.32]
Respiratory diseases	8016	2.70* [2.65–2.76]	1321	32.72* [30.98–34.53]	3355	4.46* [4.31–4.61]	3340	1.54* [1.49–1.59]
Digestive diseases	551	4.95* [4.55–5.38]	112	57.95* [47.72–69.73]	263	7.70* [6.80–8.69]	176	2.34* [2.01–2.71]
Other non–cancer diseases	17,406	2.21* [2.18–2.24]	2738	31.22* [30.06–32.41]	6839	3.95* [3.86–4.05]	7829	1.29* [1.26–1.32]

*SMR* standardized mortality ratio, *CI* confidence interval

^*^p < 0.05

^a^See Supplementary Table 1 for details

### Non-cancer causes of death at different follow-up period

Throughout the follow-up period, 55,679 (52.48%) of 106,092 patients experiencing non-cancer cause of death at a mean age of 75.84 ± 8.71 years with an overall SMR of (SMR [95% CI]) 2.42 [2.40–2.44].

#### Within 5 years after BC diagnosis

9843 (17.68%) patients died within one year after diagnosis, the mean age was 77.67 ± 8.62 with the highest overall SMR of 31.07 (95% CI [30.46–31.69]) (Fig. 3B, Tables 1, 2). The most common non-cancer cause of death was cardiovascular diseases (SMR [95% CI] 29.28 [28.47–30.12]), and other non-cancer diseases (SMR [95% CI] 31.22 [30.06–32.41]) (Table 3). While within 1–5 years after diagnosis, 22,686 (40.74%) patients experiencing non-cancer cause of death at a mean age of 77.41 ± 8.33, the overall SMR was 4.12 (95% CI [4.07–4.18]) (Fig. 3B, Tables 1, 2), cardiovascular diseases remain the most common non-cancer cause of death with a SMR of 3.98 (95% CI [3.91–4.06]) (Table 3).

The risk of death from non-cancer cause within 5 years after BC diagnosis was significantly higher than in the general population, especially for digestive diseases, infectious diseases, and diabetes mellitus (Table 3).

#### Over 5 years after BC diagnosis

25,150 (45.17%) patients died from non-cancer diseases over 5 years follow-up, the mean age was 74.09 ± 8.68, with an overall SMR of 1.35 (95% CI [1.33–1.37]) (Fig. 3B, Tables 1, 2). Cardiovascular diseases remain the most common non-cancer cause of death with a SMR of 1.30 (95% CI [1.27–1.32]) (Table 3). While patients were at significantly higher risk of death from digestive diseases (SMR [95% CI] 2.34 [2.01–2.71]) and diabetes mellitus (SMR [95% CI] 1.74 [1.62–1.86]).

### Non-cancer causes of death by different factors

Subgroup analysis of non-cancer causes of death by clinicopathological features at each follow-up period after BC diagnosis (Supplementary Tables 3–34). Some meaningful findings were as indicated below.

#### Gender

A total of 42,901 (77.05%) male patients and 12,778 (22.95%) female patients experienced non-cancer deaths, and their overall SMR was very similar (SMR [95% CI] 2.41 [2.38–2.43] vs 2.48 [2.44–2.52]). The highest proportion of deaths in male patients was observed between 1 and 5 years after BC diagnosis, and that of female patients occurred over 5 years after diagnosis. Within 1 year after diagnosis, female patients were more likely to develop infectious diseases (SMR [95% CI] 51.47 [43.38–60.63]) and diabetes mellitus (SMR [95% CI] 45.42 [36.02–56.53]) mortality, male patients were more likely to experience death from digestive diseases (SMR [95% CI] 59.19 [47.34–73.10]). And at 1–5 years after diagnosis, the causes of non-cancer mortality were similar for male and female patients, with digestive diseases being the first, followed by infectious diseases and diabetes mellitus. While over 5 years after diagnosis, male patients are at higher risk to develop digestive diseases mortality (SMR [95% CI] 2.40 [2.02–2.82]) (Supplementary Tables 5–6).

#### Race

A total of 51,269 (92.08%) white patients, 2673 (4.80%) black patients, and 1737 (3.12%) other races patients died from non-cancer diseases. The highest rate of death was observed within 1–5 years after diagnosis for blacks and more than 5 years after diagnosis for whites and other races. Whites had a higher risk of developing death from digestive diseases (SMR [95% CI] 4.91 [4.49–5.35]), while blacks had a lower risk of death from cardiovascular disease as well as other non-cancer diseases; patients of other races were at higher risk of experiencing death from infectious diseases, and diabetes mellitus. Moreover, blacks were at higher risk of experiencing death from infectious diseases within 1 year after diagnosis, and other races had a significantly higher risk of death from diabetes mellitus within 1–5 years after diagnosis. Over 5 years after BC diagnosis, blacks were at higher risk than the general population for experiencing death from infectious disease and diabetes mellitus (Supplementary Tables 7–9).

#### Treatment

Treatment is divided into surgery (TURBT: transurethral resection of bladder tumor; PC: partial cystectomy; RC: radical cystectomy), chemotherapy, and radiation therapy.

There were 2710 (4.87%) deaths in BC patients who did not receive surgical treatment, 49,655 (89.18%) deaths in patients treated with TURBT, 599 (1.08%) deaths in patients treated with PC, and 2715 (4.87%) deaths in patients treated with RC. The highest proportion of non-cancer deaths in BC patients without surgical treatment was observed 1–5 years after diagnosis (38.49%), and the highest proportion of deaths in patients who received surgical treatment all occurred 5 years after diagnosis. The risk of non-cancer causes of death was significantly higher in all BC patients compared to the general population, with the top three causes of non-cancer death being digestive disease, infectious disease and diabetes mellitus for patients who did not undergo surgery, patients who received TURBT or RC; while for patients treated with PC, the top three causes of non-cancer death were infectious disease, diabetes mellitus and cardiovascular disease. Within 1 year after diagnosis, patients treated with RC were more likely to experience non-cancer death (SMR [95% CI] 62.80 [58.39–67.44]). Although patients treated with TURBT had the lowest risk of non-cancer cause of death (SMR [95% CI] 2.36 [2.34–2.38]), it was still significantly higher than the general population (Supplementary Tables 13–16).

Patients receiving either chemotherapy (SMR [95% CI] 3.30 [3.22–3.38]) or radiation therapy (SMR [95% CI] 4.10 [3.93–4.27]) had a significantly higher risk of non-cancer deaths. Patients receiving chemotherapy appeared to be more likely to experience death from digestive (SMR [95% CI] 9.41 [7.46–11.71]) or infectious diseases (SMR [95% CI] 5.74 [5.08–6.45]), while those receiving chemotherapy had a higher risk of respiratory disease death (SMR [95% CI] 5.61 [5.03–6.24]) (Supplementary Tables 21–24).

#### Marital status

The lowest risk of non-cancer mortality was observed in widowed patients, whereas separated, divorced, and unmarried patients all had a higher risk of non-cancer mortality. Within 1 year after diagnosis, separated patients had the highest risk of non-cancer mortality (SMR [95% CI] 57.94 [42.57–77.05]), mainly from respiratory disease (SMR [95% CI] 165.13 [44.99–422.79]). Between 1 and 5 years after diagnosis, divorced patients had the highest risk of non-cancer mortality (SMR [95% CI] 6.40 [6.10–6.72]), reflected mainly in deaths from digestive and infectious diseases. Similarly, at more than 5 years after diagnosis, it was still separated patients who had the highest risk of non-cancer mortality (SMR [95% CI] 1.96 [1.86–2.05]), which was mainly represented by deaths from diabetes mellitus and digestive diseases (Supplementary Tables 25–29).

In addition, patients younger than 60 years of age had a significantly higher risk of non-cancer death than those older than 60 years of age (SMR [95% CI] 13.71 [13.22–14.22]) (Supplementary Tables 3–4). Patients diagnosed with BC in 2012–2017 had a higher risk of all non-cancer deaths than those diagnosed earlier (SMR [95% CI] 5.96 [5.84–6.07]) (Supplementary Tables 10–12). Patients with a pathological type of neuroendocrine carcinoma had the highest risk of non-cancer deaths (SMR [95% CI] 4.68 [4.06–5.37]), mainly reflected in deaths from infectious diseases (Supplementary Tables 30–34).

### Competing risk analysis

Multivariate competing risk models were used to assess prognostic factors for the development of BC-related death and non-cancer cause of death in BC patients (Table 4) and cumulative mortality (Supplementary Figs. 1 and 2). The risk of both mortalities increased significantly with increasing age, but patients of advanced age appeared to be more likely to experience non-cancer cause of death (HR [95% CI] 4.626 [4.481–4.775]) (Supplementary Figs. 1A and 2A). Female patients had a significantly lower risk of non-cancer cause of death than male patients, but their risk of BC-related death was significantly higher than that of male patients (Supplementary Figs. 1B and 2B). Black patients have the highest risk of BC-related death (HR [95% CI] 1.226 [1.180–1.274]) (Supplementary Fig. 1C). Patients who developed distant metastases had a very high risk of BC-related death (HR [95% CI] 30.345 [29.082–31.664]), in contrast to their very low risk of non-cancer cause of death (HR [95% CI] 0.429 [0.401–0.458]) (Supplementary Figs. 1D and 2D). Patients diagnosed between 2012 and 2017 had the lowest risk of non-cancer cause of death (HR [95% CI] 0.733 [0.715–0.751]) (Table 4). Patients with bladder adenocarcinoma had the lowest risk of both mortalities (Supplementary Figs. 1E and 2E). Patients who underwent RC surgery had the lowest risk of non-cancer cause of death (HR [95% CI] 0.685 [0.655–0.717]) (Supplementary Figs. 1F and 2F). The risk of non-cancer cause of death was higher in patients who did not receive either chemotherapy or radiation therapy (Supplementary Figs. 1G and 2H). Widowed patients have the highest risk of both BC-related and non-cancer cause of death (Supplementary Figs. 1I and 2I).

**Table 4:** Multivariate competing risk analysis for non–cancer diseases and bladder cancer-related hazard ratio

Factors	Cause of death			
	Bladder cancer		Non-cancer diseases	
	HR [95% CI]	*p*	HR [95% CI]	*p*
Age (years)				
00–60	Ref.		Ref.	
60+	1.469 [1.430–1.509]	< 0.05	4.626 [4.481–4.775]	< 0.05
Sex				
Male	Ref.		Ref.	
Female	1.115 [1.090–1.141]	< 0.05	0.709 [0.696–0.724]	< 0.05
Race				
White	Ref.		Ref.	
Black	1.226 [1.180–1.274]	< 0.05	0.965 [0.930–1.002]	0.063
Other races^a^	0.948 [0.902–0.996]	< 0.05	0.778 [0.744–0.813]	< 0.05
Summary stage				
In situ	Ref.		Ref.	
Localized	4.698 [4.568–4.832]	< 0.05	0.979 [0.962–0.996]	< 0.05
Regional	12.475 [11.990–12.979]	< 0.05	0.822 [0.789–0.855]	< 0.05
Distant	30.345 [29.082–31.664]	< 0.05	0.429 [0.401–0.458]	< 0.05
Year of diagnosis				
2000–2005	Ref.		Ref.	
2006–2011	1.025 [1.002–1.048]	< 0.05	0.888 [0.873–0.904]	< 0.05
2012–2017	1.002 [0.977–1.028]	0.882	0.733 [0.715–0.751]	< 0.05
Histologic type				
Tcc	Ref.		Ref.	
Scc	0.990 [0.933–1.051]	0.749	0.966 [0.911–1.025]	0.253
Nec	1.263 [1.165–1.370]	< 0.05	0.759 [0.670–0.861]	< 0.05
Ac	0.403 [0.385–0.421]	< 0.05	0.635 [0.611–0.661]	< 0.05
Oet	0.511 [0.484–0.540]	< 0.05	0.773 [0.737–0.811]	< 0.05
Surgery				
No	Ref.		Ref.	
TURBT	1.202 [1.151–1.256]	< 0.05	0.975 [0.943–1.009]	0.150
PC	0.832 [0.783–0.883]	< 0.05	0.824 [0.783–0.868]	< 0.05
RC	1.098 [1.047–1.152]	< 0.05	0.685 [0.655–0.717]	< 0.05
Radiation therapy				
Yes	Ref.		Ref.	
No/unknown	0.698 [0.676–0.721]	< 0.05	1.210 [1.165–1.257]	< 0.05
Chemotherapy				
Yes	Ref.		Ref.	
No/unknown	0.968 [0.944–0.993]	< 0.05	1.214 [1.183–1.245]	< 0.05
Marital status				
Married	Ref.		Ref.	
Separated	1.263 [1.131–1.409]	< 0.05	1.197 [1.080–1.328]	< 0.05
Divorced	1.204 [1.163–1.246]	< 0.05	1.175 [1.140–1.212]	< 0.05
Widowed	1.466 [1.427–1.507]	< 0.05	1.803 [1.766–1.840]	< 0.05
Unmarried	1.248 [1.210–1.288]	< 0.05	1.185 [1.152–1.218]	< 0.05

*CI* confidence interval, *Tcc* transitional cell carcinoma, *Scc* squamous cell carcinoma, *Nec* neuroendocrine carcinoma, *Ac* adenocarcinoma, *Oet* other epithelial tumors, *TURBT* transurethral resection of bladder tumor, *PC* partial cystectomy, *RC* radical cystectomy

^a^Including American Indian/Alaska Native and Asian or Pacific Islander

## Discussion

Bladder cancer as one of the most common malignancies in urology, patients are often more concerned about BC-related mortality. However, due to continuous medical advances and developments, the overall survival of BC patients has been effectively improved(Richters et al. 2020; Lenis et al. 2020; Marrie et al. 2021), leading to the prominence of deaths from non-cancer diseases (Wang et al. 2022a).

Our study found that non-cancer causes of death from BC accounted for 52.48% of all deaths, with cardiovascular disease being the most common non-cancer cause of death, followed by respiratory disease, diabetes mellitus, and infectious diseases. Published studies showed that cardiovascular disease is one of the leading causes of non-cancer mortality (Du et al. 2021; Oh et al. 2020). Studies show that cancer patients are 2–6 times more likely to die from cardiovascular disease than the general population (Sturgeon et al. 2019). Studies have shown that chemotherapy and radiation therapy for cancer increase heart disease-specific mortality in patients (Yang et al. 2021; Dai et al. 2022; Guan et al. 2021). Meanwhile, other non-cancer diseases such as diabetes mellitus and nephrotic syndrome increase the cardiac burden by altering the body's metabolic status and hemodynamics. The risk of cardiovascular disease mortality in BC patients continues throughout the treatment and follow-up period. Therefore, we recommend more proactive self-observation and follow-up rather than reactive management only at the onset of clinical symptoms or complications, and that such self-observation and follow-up should continue from the start of oncologic treatment and for the rest of the life.

The incidence of respiratory disease is higher among cancer patients (Li et al. 2020; Deckx et al. 2012). Among them, pulmonary infections caused by neutropenia are the most common. Published meta-analysis showed that the use of immune checkpoint inhibitors, compared to chemotherapy, significantly increased the incidence of pneumonia (Nishijima et al. 2017). Also, the incidence of chronic obstructive pulmonary disease was significantly higher in cancer patients (Song et al. 2022; Zhang et al. 2021). Also, the increase in smoking and air pollution has led to an increase in the incidence of pneumonia or chronic obstructive pulmonary disease in cancer patients.

In cancer patients, it is very common for antitumor therapy (e.g., chemotherapy) to cause myelosuppression (Simonaggio et al. 2019). Neutropenia is an independent risk factor for sepsis (Kochanek et al. 2019b). Some studies have shown that cancer patients who have developed sepsis or bacteremia have an approximately 2.3 times higher risk of death than non-cancer patients (Abou Dagher et al. 2017). In addition, Liyanage et al. (2002) and Schreiber et al. (2011) showed that tumors can also cause an increased risk of infection in cancer patients through metastatic invasion and mechanical damage to the immune system.

The relationship between BC and diabetes mellitus should not be underestimated. Patients with a long history of impaired fasting glucose or diabetes mellitus are at significantly higher risk of BC due to diet, obesity and other factors (Choi et al. 2022; Lam et al. 2021; Gill et al. 2021). A meta-analysis suggests that diabetes mellitus has an increased risk of disease progression, recurrence and death in BC patients (Lu and Tao 2021). Meanwhile, our study showed a significantly higher SMR for diabetes mellitus -related deaths among BC patients, consistent with published studies. In addition, some studies have suggested that metformin use is associated with a better prognosis in non-muscle invasive BC (Wang et al. 2022b; Liu et al. 2022; Klose et al. 2021), but larger population studies are needed to confirm this.

Multivariate competing risk analysis showed that patients with BC are at significantly higher risk of experiencing death from non-cancer causes compared to the general population, with potential risk factors including: age > 60 years, male, whites, in situ stage, pathological type of transitional cell carcinoma, not receiving treatment (including surgery, chemotherapy, or radiation), and widowed. Therefore, for high-risk patients, we recommend aggressive treatment and follow-up. It is estimated that primary prevention of 30–40% of cancers can be achieved by modifying lifestyle and environmental risk factors known to be associated with cancer incidence. A healthy lifestyle, including reducing smoking, low BMI, being physically active, avoiding excessive alcohol consumption and maintaining a healthy nutrition are the best strategies to prevent and treat non-cancer diseases (Zhang et al. 2020).

Our study is limited by the inherent bias of the SEER database and the fact that this is a retrospective study without external validation. We reduce bias due to factors such as age, sex, and race by strictly controlling inclusion criteria and using SMR, while quantitatively assessing the impact of different factors on non-cancer mortality in BC patients through multivariate competing risk analysis. However, our inability to know whether patients had relevant non-cancerous diseases before diagnosis and the lack of data on some high-risk factors, such as smoking, alcohol consumption, and lifestyle, led to certain shortcomings in our study. Therefore, future high-quality studies are needed to explore the major causes of non-cancer mortality in BC patients and the associated risk factors.

## Conclusion

Non-cancer diseases accounted for the highest proportion of deaths during each follow-up period after BC diagnosis, with cardiovascular diseases being the most common causes. SMR for non-cancer causes of death decreased progressively with increasing follow-up time and was approximately 7.5 times higher within 1 year than 1–5 years after diagnosis. Additionally, the risk of non-cancer causes of death in BC patients is impacted by a number of factors that are inherently heterogeneous, such as age, gender, treatment modality (surgery, chemotherapy, and radiation therapy), and marital status. Therefore, physicians should pay attention to the risk of death from non-cancer diseases. Also, physicians should encourage patients to engage in more proactive self-surveillance and follow up.

## Supplementary Information

Below is the link to the electronic supplementary material.

Supplementary file1 Supplementary figure 1 Cumulative mortality curves for bladder cancer-related deaths in patients with bladder cancer stratified by age (A), sex (B), race (C), summary stage (D), histologic type (E), surgery (F), chemotherapy (G), radiation therapy (H), and marital status (I) (TIF 7240 KB)

Supplementary file2 Supplementary figure 2 Cumulative mortality curves for non-cancer deaths in patients with bladder cancer stratified by age (A), sex (B), race (C), summary stage (D), histologic type (E), surgery (F), chemotherapy (G), radiation therapy (H), and marital status (I) (TIF 6596 KB)

Supplementary file3 (DOCX 17 KB)

Supplementary file4 (DOCX 28 KB)

Supplementary file5 (DOCX 100 KB)

## Data Availability

The data in this study were obtained from the publicly available SEER database (https://seer.cancer.gov/: Incidence—SEER Research Plus Data, 18 Registries (excl AK), Nov 2020 Sub (2000–2018) for SMRs).

## Abbreviations

BC: Bladder cancer
SEER: The Surveillance, Epidemiology, and End Results
SMR: Standardized mortality ratio
CI: Confidence interval
Tcc: Transitional cell carcinoma
Scc: Squamous cell carcinoma
Nec: Neuroendocrine carcinoma
Ac: Adenocarcinoma
Oet: Other epithelial tumors
TURBT: Transurethral resection of bladder tumor
PC: Partial cystectomy
RC: Radical cystectomy
