# Supplementary figures and images for: High risk of non-cancer mortality in bladder cancer patients: evidence from SEER-Medicaid
Source: J Cancer Res Clin Oncol. 2023 Jun 3;149(12):10203–15. doi: 10.1007/s00432-023-04867-z (PMC10423154; doi:10.1007/s00432-023-04867-z)

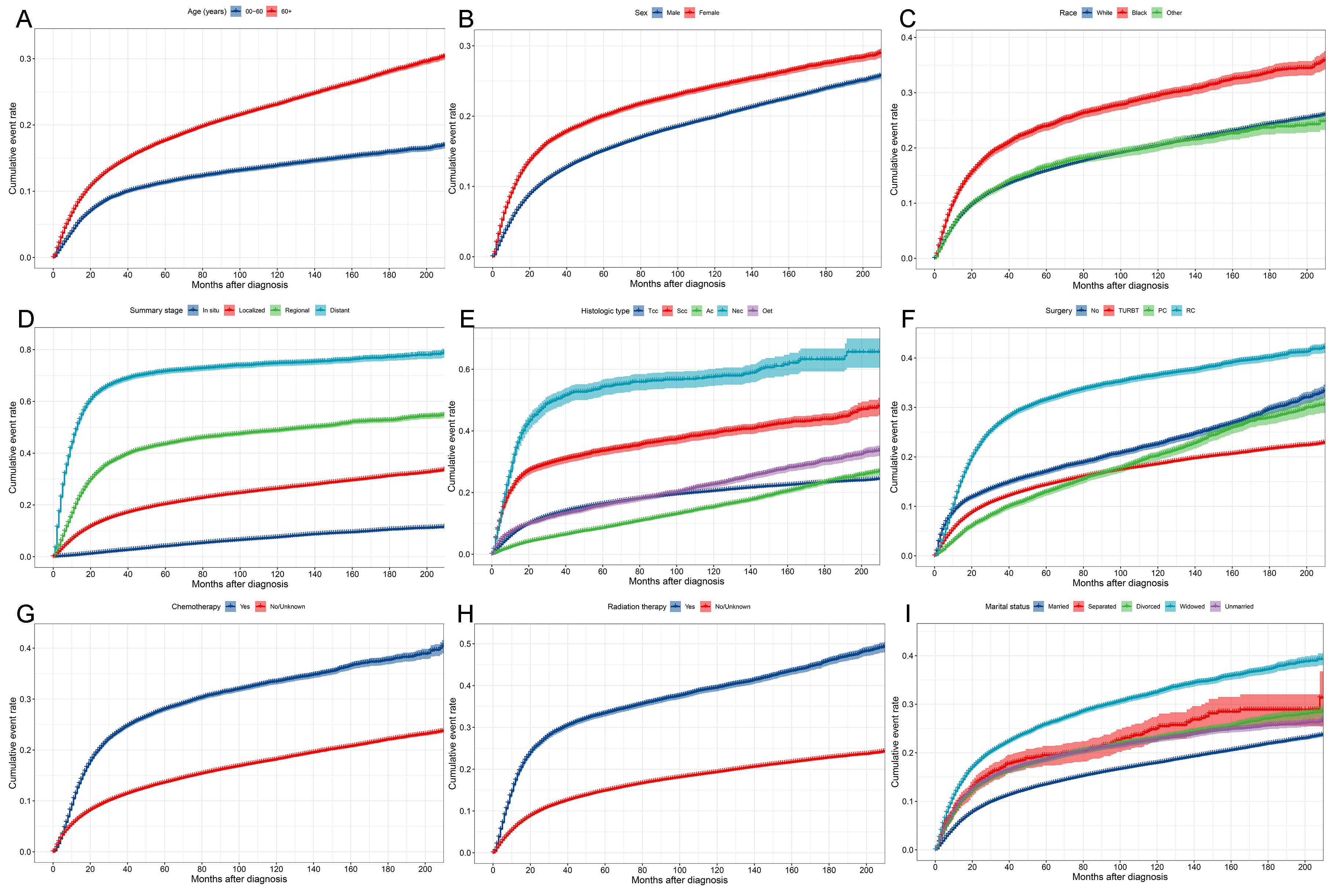

Supplement: Supplementary file 1 — Supplementary file1 Supplementary figure 1 Cumulative mortality curves for bladder cancer-related deaths in patients with bladder cancer stratified by age (A), sex (B), race (C), summary stage (D), histologic type (E), surgery (F), chemotherapy (G), radiation therapy (H), and marital status (I) (TIF 7240 KB) [file 432_2023_4867_MOESM1_ESM.tif]

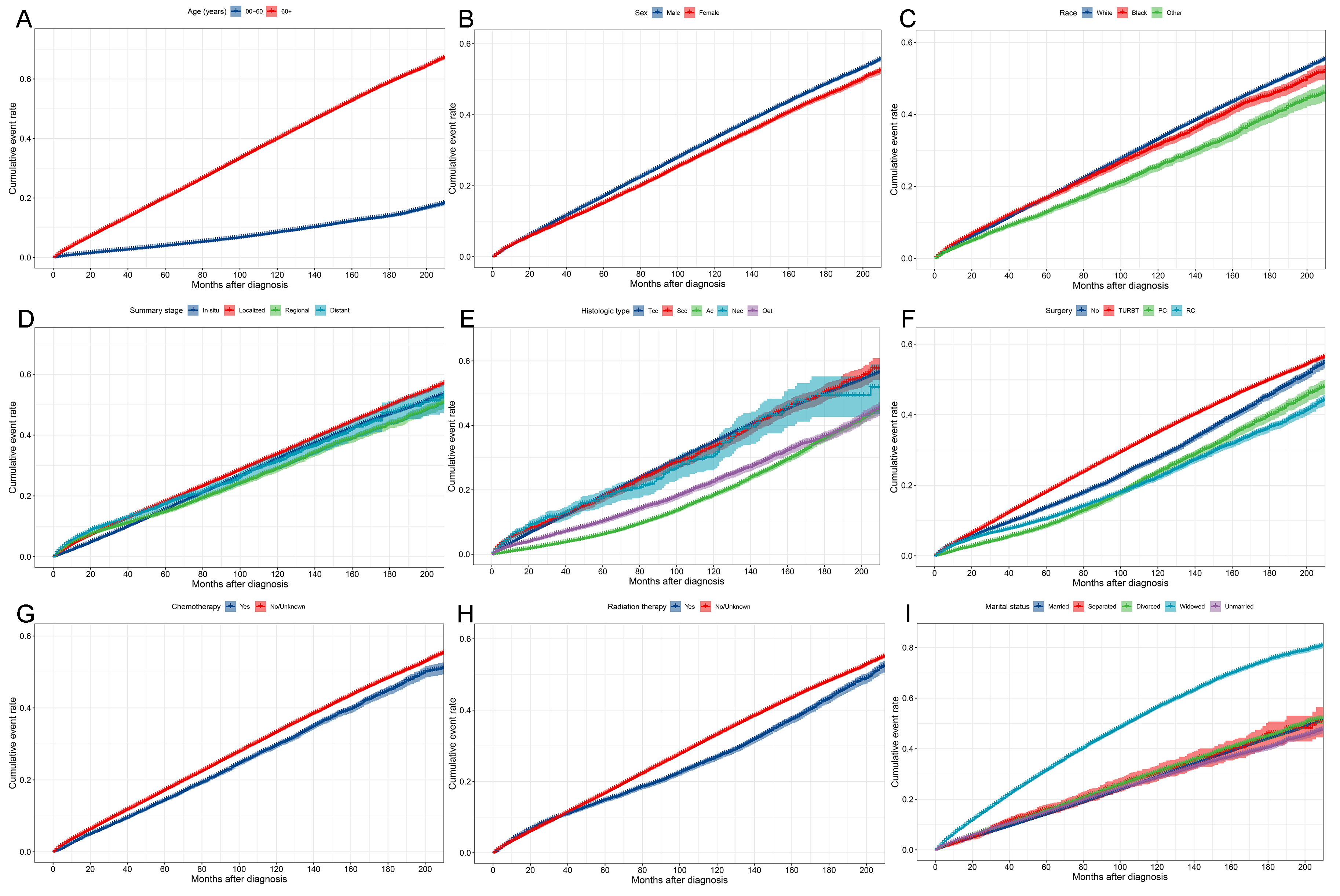

Supplement: Supplementary file 2 — Supplementary file2 Supplementary figure 2 Cumulative mortality curves for non-cancer deaths in patients with bladder cancer stratified by age (A), sex (B), race (C), summary stage (D), histologic type (E), surgery (F), chemotherapy (G), radiation therapy (H), and marital status (I) (TIF 6596 KB) [file 432_2023_4867_MOESM2_ESM.tif]
