# Supplementary material for: High risk of non-cancer mortality in bladder cancer patients: evidence from SEER-Medicaid
Source: J Cancer Res Clin Oncol. 2023 Jun 3;149(12):10203–15. doi: 10.1007/s00432-023-04867-z (PMC10423154; doi:10.1007/s00432-023-04867-z)
Supplement: Supplementary file 3 — Supplementary file3 (DOCX 17 KB) [file 432_2023_4867_MOESM3_ESM.docx]

| **Supplementary Table 1** Definition of non–cancer causes of death and ICD–10 codes of diseases | | |
| --- | --- | --- |
| Non–cancer causes of death | ICD–10 codes | Causes of death definition |
| Infectious diseases | A15–A19 | 1. Tuberculosis |
|  | A50–A53 | 1. Syphilis |
|  | A40–A41 | 1. Septicemia |
|  | A00–A08, A20–A33, A35–A39, A40–A49, A54–B19, B25–B99 | 1. Other Infectious and Parasitic Diseases including HIV |
| Diabetes Mellitus | E10–E14 | 1. Diabetes Mellitus |
| Cardiovascular diseases | I00–I09, I11, I13, I20–I51 | 1. Diseases of Heart |
|  | I10, I12 | 1. Hypertension without Heart Disease |
|  | I60–I69 | 1. Cerebrovascular Diseases |
|  | I70 | 1. Atherosclerosis |
|  | I71 | 1. Aortic Aneurysm and Dissection |
|  | I72–I78 | 1. Other Diseases of Arteries, Arterioles, Capillaries |
| Respiratory diseases | J09–J18 | 1. Pneumonia and Influenza |
|  | J40–J47 | 1. Chronic Obstructive Pulmonary Disease and Allied Cond |
| Digestive diseases | K25–K28 | 1. Stomach and Duodenal Ulcers |
|  | K70, K73–K74 | 1. Chronic Liver Disease and Cirrhosis |
| Other non–cancer diseases | N00–N07, N17–N19, N25–N27 | 1. Nephritis, Nephrotic Syndrome and Nephrosis |
|  | G30 | 1. Alzheimer |
|  | A34, O00–O95, O98–O99 | 1. Complications of Pregnancy, Childbirth, Puerperium |
|  | Q00–Q99 | 1. Congenital Anomalies |
|  | P00–P96 | 1. Certain Conditions Originating in Perinatal Period |
|  | R00–R99 | 1. Symptoms, Signs and Ill–Defined Conditions |
|  | V01–X59, Y85–Y86 | 1. Accidents and Adverse Effects |
|  | U03, X60–X84, Y87.0 | 1. Suicide and Self–Inflicted Injury |
|  | U01–U02, X85–Y09, Y35, Y87.1, Y89.0 | 1. Homicide and Legal Intervention |
|  | – | 1. Other Cause of Death |
