# Supplementary material for: High risk of non-cancer mortality in bladder cancer patients: evidence from SEER-Medicaid
Source: J Cancer Res Clin Oncol. 2023 Jun 3;149(12):10203–15. doi: 10.1007/s00432-023-04867-z (PMC10423154; doi:10.1007/s00432-023-04867-z)
Supplement: Supplementary file 4 — Supplementary file4 (DOCX 28 KB) [file 432_2023_4867_MOESM4_ESM.docx]

| **Supplementary Table 2** SMR for all causes of death in bladder cancer patients after diagnosis | | | | | | | | |
| --- | --- | --- | --- | --- | --- | --- | --- | --- |
| Factors | Overall | | Causes of death | | | | | |
|  |  |  | Bladder cancer | | Other cancers | | Non–cancer diseases | |
|  | Observed | SMR [95% CI] | Observed | SMR [95% CI] | Observed | SMR [95% CI] | Observed | SMR [95% CI] |
| Total | 106092 | 3.23*[3.21–3.25] | 37205 | 7.59*[7.52–7.67] | 13208 | 2.68*[2.63–2.72] | 55679 | 2.42*[2.40–2.44] |
| Age (years) |  |  |  |  |  |  |  |  |
| 00–60 | 9003 | 21.10*[20.66–21.54] | 4871 | 48.48*[47.13–49.87] | 1256 | 10.78*[10.19–11.39] | 2876 | 13.71*[13.22–14.22] |
| 60+ | 97089 | 3.00*[2.98–3.02] | 32334 | 6.74*[6.66–6.81] | 11952 | 2.48*[2.44–2.53] | 52803 | 2.32*[2.30–2.34] |
| Sex |  |  |  |  |  |  |  |  |
| Male | 78547 | 3.06*[3.04–3.08] | 25582 | 6.79*[6.71–6.88] | 10064 | 2.47*[2.42–2.52] | 42901 | 2.41*[2.38–2.43] |
| Female | 27545 | 3.85*[3.81–3.90] | 11623 | 10.26*[10.07–10.45] | 3144 | 3.65*[3.52–3.78] | 12778 | 2.48*[2.44–2.52] |
| Race |  |  |  |  |  |  |  |  |
| White | 95627 | 3.12*[3.10–3.14] | 32629 | 7.28*[7.20–7.36] | 11729 | 2.57*[2.52–2.62] | 51269 | 2.37*[2.35–2.39] |
| Black | 6671 | 4.87*[4.75–4.98] | 3020 | 10.50*[10.13–10.88] | 978 | 3.88*[3.64–4.13] | 2673 | 3.22*[3.10–3.34] |
| Other races^#^ | 3794 | 5.00*[4.84–5.16] | 1556 | 11.87*[11.28–12.47] | 501 | 4.26*[3.89–4.65] | 1737 | 3.40*[3.24–3.57] |
| Summary stage |  |  |  |  |  |  |  |  |
| In situ | 39351 | 2.08*[2.05–2.10] | 3433 | 2.05*[1.98–2.12] | 5163 | 1.76*[1.71–1.81] | 30755 | 2.14*[2.12–2.17] |
| Localized | 46094 | 3.72*[3.69–3.76] | 19037 | 7.28*[7.17–7.38] | 5372 | 3.04*[2.95–3.12] | 21685 | 2.71*[2.68–2.75] |
| Regional | 12098 | 9.99*[9.81–10.17] | 8143 | 17.92*[17.53–18.31] | 1425 | 7.85*[7.45–8.27] | 2530 | 4.40*[4.23–4.57] |
| Distant | 8549 | 32.90*[32.21–33.61] | 6592 | 42.21*[41.20–43.25] | 1248 | 29.64*[28.02–31.33] | 709 | 11.52*[10.69–12.4] |
| Year of diagnosis |  |  |  |  |  |  |  |  |
| 2000–2005 | 44445 | 2.31*[2.29–2.33] | 12859 | 5.56*[5.46–5.65] | 5261 | 1.83*[1.78–1.88] | 26325 | 1.88*[1.85–1.90] |
| 2006–2011 | 37393 | 3.49*[3.45–3.52] | 13058 | 7.32*[7.19–7.45] | 4715 | 2.91*[2.82–2.99] | 19620 | 2.68*[2.65–2.72] |
| 2012–2017 | 24254 | 8.47*[8.36–8.57] | 11288 | 14.09*[13.83–14.36] | 3232 | 7.53*[7.27–7.79] | 9734 | 5.96*[5.84–6.07] |
| Histologic type |  |  |  |  |  |  |  |  |
| Tcc | 98685 | 3.10*[3.08–3.12] | 32887 | 7.04*[6.97–7.12] | 12126 | 2.53*[2.48–2.57] | 53672 | 2.40*[2.38–2.42] |
| Scc | 2704 | 6.81*[6.55–7.07] | 1592 | 20.14*[19.16–21.15] | 308 | 5.07*[4.49–5.70] | 804 | 3.11*[2.90–3.33] |
| Nec | 1329 | 13.63*[12.89–14.40] | 914 | 24.00*[22.44–25.65] | 202 | 14.40*[12.43–16.60] | 213 | 4.68*[4.06–5.37] |
| Ac | 1538 | 9.14*[8.68–9.62] | 909 | 19.38*[18.13–20.70] | 267 | 11.80*[10.40–13.33] | 362 | 3.66*[3.29–4.06] |
| Oet | 1836 | 6.08*[5.81–6.37] | 903 | 13.48*[12.60–14.40] | 305 | 6.91*[6.15–7.75] | 628 | 3.31*[3.05–3.58] |
| Surgery |  |  |  |  |  |  |  |  |
| No | 6845 | 4.95*[4.83–5.07] | 2872 | 12.83*[12.37–13.31] | 1263 | 5.71*[5.40–6.04] | 2710 | 2.89*[2.78–3.00] |
| TURBT | 86765 | 2.92*[2.91–2.94] | 26542 | 6.34*[6.26–6.42] | 10568 | 2.38*[2.34–2.43] | 49655 | 2.36*[2.34–2.38] |
| PC | 1740 | 4.33*[4.13–4.54] | 931 | 10.41*[9.75–11.10] | 210 | 2.74*[2.38–3.13] | 599 | 2.54*[2.34–2.76] |
| RC | 10742 | 7.86*[7.71–8.01] | 6860 | 17.13*[16.73–17.54] | 1167 | 5.96*[5.63–6.31] | 2715 | 3.52*[3.39–3.66] |
| Radiation therapy |  |  |  |  |  |  |  |  |
| Yes | 9473 | 7.78*[7.62–7.94] | 6104 | 12.53*[12.21–12.84] | 1024 | 6.48*[6.09–6.89] | 2345 | 4.10*[3.93–4.27] |
| No/Unknown | 96619 | 3.06*[3.04–3.08] | 31101 | 7.05*[6.97–7.13] | 12184 | 2.55*[2.51–2.60] | 53334 | 2.38*[2.36–2.40] |
| Chemotherapy |  |  |  |  |  |  |  |  |
| Yes | 21242 | 6.18*[6.10–6.26] | 12031 | 13.32*[13.09–13.57] | 2509 | 4.96*[4.77–5.16] | 6702 | 3.30*[3.22–3.38] |
| No/Unknown | 84850 | 2.89*[2.87–2.91] | 25174 | 6.30*[6.22–6.38] | 10699 | 2.42*[2.37–2.46] | 48977 | 2.34*[2.32–2.36] |
| Marital status |  |  |  |  |  |  |  |  |
| Married | 61023 | 2.95*[2.93–2.97] | 20064 | 6.80*[6.71–6.89] | 7967 | 2.38*[2.33–2.44] | 32992 | 2.29*[2.27–2.32] |
| Separated | 717 | 5.08*[4.71–5.46] | 298 | 13.94*[12.40–15.62] | 96 | 4.20*[3.40–5.13] | 323 | 3.33*[2.98–3.71] |
| Divorced | 8500 | 5.34*[5.23–5.46] | 3438 | 13.36*[12.91–13.81] | 1138 | 4.32*[4.07–4.58] | 3924 | 3.66*[3.55–3.78] |
| Widowed | 24905 | 3.00*[2.97–3.04] | 8755 | 6.56*[6.43–6.70] | 2653 | 2.70*[2.59–2.80] | 13497 | 2.26*[2.22–2.30] |
| Unmarried | 10947 | 5.17*[5.07–5.27] | 4650 | 13.85*[13.46–14.25] | 1354 | 4.20*[3.98–4.43] | 4943 | 3.39*[3.29–3.48] |

Abbreviation: SMR: standardized mortality ratio; CI: confidence interval; Tcc: transitional cell carcinoma; Scc: squamous cell carcinoma; Nec: neuroendocrine carcinoma; Ac: adenocarcinoma; Oet: other epithelial tumors; TURBT: transurethral resection of bladder tumor; PC: partial cystectomy; RC: radical cystectomy

**p*<0.05

^#^ Including American Indian/Alaska Native and Asian or Pacific Islander.
