# Supplementary material for: High risk of non-cancer mortality in bladder cancer patients: evidence from SEER-Medicaid
Source: J Cancer Res Clin Oncol. 2023 Jun 3;149(12):10203–15. doi: 10.1007/s00432-023-04867-z (PMC10423154; doi:10.1007/s00432-023-04867-z)
Supplement: Supplementary file 5 — Supplementary file5 (DOCX 100 KB) [file 432_2023_4867_MOESM5_ESM.docx]

| **Supplementary Table 3** SMR for non–cancer causes of death following bladder cancer diagnosis in patients aged younger than 60 years | | | | | | | | |
| --- | --- | --- | --- | --- | --- | --- | --- | --- |
| Non–cancer diseases^#^ | Overall | | <1 year | | 1–5 years | | >5 years | |
|  | Observed | SMR [95% CI] | Observed | SMR [95% CI] | Observed | SMR [95% CI] | Observed | SMR [95% CI] |
| Total | 2876 | 13.71*[13.22–14.22] | 421 | 351.91*[319.09–387.18] | 945 | 40.62*[38.08–43.30] | 1510 | 8.15*[7.74–8.57] |
| Infectious diseases | 161 | 22.40*[19.07–26.13] | 49 | 463.31*[342.76–612.53] | 45 | 45.47*[33.16–60.84] | 67 | 11.00*[8.52–13.96] |
| Diabetes Mellitus | 151 | 14.34*[12.14–16.82] | 12 | 322.22*[166.50–562.86] | 57 | 38.47*[29.13–49.84] | 82 | 9.10*[7.24–11.29] |
| Cardiovascular diseases | 1134 | 12.42*[11.70–13.16] | 161 | 308.54*[262.72–360.05] | 346 | 37.37*[33.53–41.52] | 627 | 7.69*[7.10–8.31] |
| Respiratory diseases | 341 | 10.53*[9.44–11.71] | 28 | 307.58*[204.38–444.54] | 105 | 36.76*[30.07–44.51] | 208 | 7.07*[6.14–8.10] |
| Digestive diseases | 103 | 22.53*[18.39–27.33] | 20 | 536.67*[327.81–828.84] | 44 | 44.25*[32.15–59.41] | 39 | 11.02*[7.84–15.06] |
| Other non–cancer diseases | 986 | 15.47*[14.52–16.47] | 151 | 374.48*[317.14–439.20] | 348 | 45.31*[40.68–50.33] | 487 | 8.75*[7.99–9.56] |

Abbreviation: SMR: standardized mortality ratio; CI: confidence interval

**p*<0.05

^#^See **Supplementary Table 1** for details.

| **Supplementary Table 4** SMR for non–cancer causes of death following bladder cancer diagnosis in patients aged over 60 years | | | | | | | | |
| --- | --- | --- | --- | --- | --- | --- | --- | --- |
| Non–cancer diseases^#^ | Overall | | <1 year | | 1–5 years | | >5 years | |
|  | Observed | SMR [95% CI] | Observed | SMR [95% CI] | Observed | SMR [95% CI] | Observed | SMR [95% CI] |
| Total | 52803 | 2.32*[2.30–2.34] | 9422 | 29.85*[29.25–30.46] | 21741 | 3.97*[3.91–4.02] | 21640 | 1.27*[1.26–1.29] |
| Infectious diseases | 1567 | 3.06*[2.91–3.21] | 423 | 36.20*[32.83–39.81] | 611 | 4.89*[4.51–5.30] | 533 | 1.42*[1.30–1.54] |
| Diabetes Mellitus | 1846 | 2.92*[2.79–3.06] | 341 | 35.98*[32.26–40.01] | 768 | 4.80*[4.46–5.15] | 737 | 1.60*[1.48–1.71] |
| Cardiovascular diseases | 24847 | 2.31*[2.28–2.33] | 4686 | 28.40*[27.59–29.23] | 10402 | 3.87*[3.79–3.94] | 9759 | 1.23*[1.21–1.26] |
| Respiratory diseases | 7675 | 2.62*[2.56–2.68] | 1293 | 32.10*[30.37–33.90] | 3250 | 4.33*[4.19–4.49] | 3132 | 1.46*[1.41–1.52] |
| Digestive diseases | 448 | 4.20*[3.82–4.61] | 92 | 48.54*[39.13–59.53] | 219 | 6.60*[5.76–7.54] | 137 | 1.91*[1.61–2.26] |
| Other non–cancer diseases | 16420 | 2.10*[2.07–2.13] | 2587 | 29.63*[28.50–30.80] | 6491 | 3.77*[3.68–3.86] | 7342 | 1.22*[1.19–1.25] |

Abbreviation: SMR: standardized mortality ratio; CI: confidence interval

**p*<0.05

^#^See **Supplementary Table 1** for details.

| **Supplementary Table 5** SMR for non–cancer causes of death following bladder cancer diagnosis in male patients | | | | | | | | |
| --- | --- | --- | --- | --- | --- | --- | --- | --- |
| Non–cancer diseases^#^ | Overall | | <1 year | | 1–5 years | | >5 years | |
|  | Observed | SMR [95% CI] | Observed | SMR [95% CI] | Observed | SMR [95% CI] | Observed | SMR [95% CI] |
| Total | 42901 | 2.41*[2.38–2.43] | 7521 | 30.07*[29.39–30.76] | 17948 | 4.04*[3.98–4.10] | 17432 | 1.33*[1.31–1.35] |
| Infectious diseases | 1259 | 3.21*[3.03–3.39] | 329 | 36.50*[32.66–40.66] | 508 | 5.17*[4.73–5.65] | 422 | 1.48*[1.34–1.63] |
| Diabetes Mellitus | 1618 | 3.07*[2.92–3.22] | 273 | 35.21*[31.16–39.64] | 691 | 5.06*[4.69–5.45] | 654 | 1.71*[1.58–1.84] |
| Cardiovascular diseases | 20329 | 2.38*[2.34–2.41] | 3755 | 28.72*[27.81–29.66] | 8579 | 3.91*[3.82–3.99] | 7995 | 1.28*[1.26–1.31] |
| Respiratory diseases | 6048 | 2.61*[2.54–2.67] | 1005 | 31.45*[29.53–33.45] | 2568 | 4.32*[4.16–4.49] | 2475 | 1.46*[1.40–1.52] |
| Digestive diseases | 451 | 4.95*[4.50–5.42] | 86 | 59.19*[47.34–73.10] | 221 | 7.44*[6.49–8.49] | 144 | 2.40*[2.02–2.82] |
| Other non–cancer diseases | 13196 | 2.22*[2.18–2.26] | 2073 | 29.95*[28.67–31.27] | 5381 | 3.88*[3.78–3.99] | 5742 | 1.28*[1.24–1.31] |

Abbreviation: SMR: standardized mortality ratio; CI: confidence interval

**p*<0.05

^#^See **Supplementary Table 1** for details.

| **Supplementary Table 6** SMR for non–cancer causes of death following bladder cancer diagnosis in female patients | | | | | | | | |
| --- | --- | --- | --- | --- | --- | --- | --- | --- |
| Non–cancer diseases^#^ | Overall | | <1 year | | 1–5 years | | >5 years | |
|  | Observed | SMR [95% CI] | Observed | SMR [95% CI] | Observed | SMR [95% CI] | Observed | SMR [95% CI] |
| Total | 12778 | 2.48*[2.44–2.52] | 2322 | 34.81*[33.41–36.25] | 4738 | 4.45*[4.33–4.58] | 5718 | 1.42*[1.39–1.46] |
| Infectious diseases | 469 | 3.69*[3.36–4.04] | 143 | 51.47*[43.38–60.63] | 148 | 5.35*[4.52–6.29] | 178 | 1.84*[1.58–2.13] |
| Diabetes Mellitus | 379 | 3.31*[2.99–3.66] | 80 | 45.42*[36.02–56.53] | 134 | 5.38*[4.51–6.37] | 165 | 1.88*[1.61–2.19] |
| Cardiovascular diseases | 5652 | 2.44*[2.38–2.51] | 1092 | 31.39*[29.56–33.31] | 2169 | 4.30*[4.12–4.48] | 2391 | 1.35*[1.29–1.40] |
| Respiratory diseases | 1968 | 3.06*[2.93–3.20] | 316 | 37.55*[33.52–41.92] | 787 | 4.95*[4.61–5.31] | 865 | 1.82*[1.70–1.94] |
| Digestive diseases | 100 | 4.98*[4.06–6.06] | 26 | 54.21*[35.41–79.43] | 42 | 9.40*[6.78–12.71] | 32 | 2.12*[1.45–2.99] |
| Other non–cancer diseases | 4210 | 2.18*[2.11–2.25] | 665 | 35.97*[33.29–38.81] | 1458 | 4.24*[4.02–4.46] | 2087 | 1.33*[1.27–1.39] |

Abbreviation: SMR: standardized mortality ratio; CI: confidence interval

**p*<0.05

^#^See **Supplementary Table 1** for details.

| **Supplementary Table 7** SMR for non–cancer causes of death following bladder cancer diagnosis in white patients | | | | | | | | |
| --- | --- | --- | --- | --- | --- | --- | --- | --- |
| Non–cancer diseases^#^ | Overall | | <1 year | | 1–5 years | | >5 years | |
|  | Observed | SMR [95% CI] | Observed | SMR [95% CI] | Observed | SMR [95% CI] | Observed | SMR [95% CI] |
| Total | 51269 | 2.37*[2.35–2.39] | 8873 | 30.18*[29.56–30.82] | 20914 | 4.03*[3.98–4.08] | 21482 | 1.33*[1.31–1.35] |
| Infectious diseases | 1527 | 3.21*[3.05–3.38] | 408 | 38.19*[34.58–42.08] | 581 | 4.98*[4.59–5.41] | 538 | 1.55*[1.42–1.68] |
| Diabetes Mellitus | 1762 | 3.04*[2.90–3.19] | 306 | 35.89*[31.98–40.14] | 729 | 4.96*[4.61–5.34] | 727 | 1.72*[1.59–1.85] |
| Cardiovascular diseases | 23886 | 2.33*[2.30–2.36] | 4334 | 28.41*[27.57–29.27] | 9915 | 3.89*[3.81–3.96] | 9637 | 1.28*[1.25–1.30] |
| Respiratory diseases | 7517 | 2.68*[2.62–2.74] | 1242 | 32.54*[30.76–34.41] | 3138 | 4.38*[4.23–4.54] | 3137 | 1.53*[1.48–1.58] |
| Digestive diseases | 516 | 4.91*[4.49–5.35] | 107 | 58.13*[47.64–70.25] | 246 | 7.58*[6.66–8.58] | 163 | 2.30*[1.96–2.68] |
| Other non–cancer diseases | 16061 | 2.16*[2.13–2.19] | 2476 | 30.11*[28.93–31.32] | 6305 | 3.88*[3.78–3.97] | 7280 | 1.27*[1.24–1.30] |

Abbreviation: SMR: standardized mortality ratio; CI: confidence interval

**p*<0.05

^#^See **Supplementary Table 1** for details.

| **Supplementary Table 8** SMR for non–cancer causes of death following bladder cancer diagnosis in black patients | | | | | | | | |
| --- | --- | --- | --- | --- | --- | --- | --- | --- |
| Non–cancer diseases^#^ | Overall | | <1 year | | 1–5 years | | >5 years | |
|  | Observed | SMR [95% CI] | Observed | SMR [95% CI] | Observed | SMR [95% CI] | Observed | SMR [95% CI] |
| Total | 2673 | 3.22*[3.10–3.34] | 621 | 38.49*[35.52–41.64] | 1104 | 5.30*[5.00–5.63] | 948 | 1.56*[1.46–1.67] |
| Infectious diseases | 157 | 4.57*[3.88–5.34] | 50 | 60.43*[44.85–79.67] | 63 | 7.49*[5.75–9.58] | 44 | 1.75*[1.27–2.35] |
| Diabetes Mellitus | 141 | 3.46*[2.91–4.08] | 28 | 35.03*[23.28–50.63] | 57 | 5.85*[4.43–7.58] | 56 | 1.85*[1.40–2.40] |
| Cardiovascular diseases | 1283 | 3.31*[3.14–3.50] | 329 | 35.99*[32.20–40.10] | 520 | 5.25*[4.81–5.72] | 434 | 1.56*[1.41–1.71] |
| Respiratory diseases | 255 | 3.15*[2.77–3.56] | 38 | 27.11*[19.19–37.21] | 120 | 5.98*[4.96–7.16] | 97 | 1.63*[1.32–1.99] |
| Digestive diseases | 20 | 5.52*[3.37–8.53] | 4 | 47.59*[12.97–121.85] | 9 | 12.42*[5.68–23.58] | 7 | 2.49*[1.00–5.13] |
| Other non–cancer diseases | 817 | 2.88*[2.68–3.08] | 172 | 44.34*[37.96–51.49] | 335 | 4.78*[4.28–5.31] | 310 | 1.48*[1.32–1.65] |

Abbreviation: SMR: standardized mortality ratio; CI: confidence interval

**p*<0.05

^#^See **Supplementary Table 1** for details.

| **Supplementary Table 9** SMR for non–cancer causes of death following bladder cancer diagnosis in patients of other races | | | | | | | | |
| --- | --- | --- | --- | --- | --- | --- | --- | --- |
| Non–cancer diseases^#^ | Overall | | <1 year | | 1–5 years | | >5 years | |
|  | Observed | SMR [95% CI] | Observed | SMR [95% CI] | Observed | SMR [95% CI] | Observed | SMR [95% CI] |
| Total | 1737 | 3.40*[3.24–3.57] | 349 | 52.07*[46.75–57.83] | 668 | 6.28*[5.82–6.78] | 720 | 1.81*[1.68–1.95] |
| Infectious diseases | 44 | 4.40*[3.19–5.90] | 14 | 49.61*[27.12–83.24] | 12 | 14.28*[7.38–24.94] | 18 | 2.03*[1.20–3.20] |
| Diabetes Mellitus | 94 | 4.25*[3.43–5.20] | 19 | 100.53*[60.53–156.99] | 39 | 7.99*[5.68–10.92] | 36 | 2.11*[1.48–2.92] |
| Cardiovascular diseases | 812 | 3.51*[3.27–3.76] | 184 | 48.10*[41.40–55.57] | 313 | 6.33*[5.65–7.07] | 315 | 1.77*[1.58–1.98] |
| Respiratory diseases | 244 | 3.13*[2.75–3.55] | 41 | 50.66*[36.36–68.73] | 97 | 5.68*[4.60–6.92] | 106 | 1.76*[1.44–2.13] |
| Digestive diseases | 15 | 6.10*[3.41–10.06] | 1 | 126.85*[3.21–706.75] | 8 | 8.34*[3.60–16.43] | 6 | 4.02*[1.48–8.76] |
| Other non–cancer diseases | 528 | 3.17*[2.90–3.45] | 90 | 56.66*[45.56–69.64] | 199 | 6.01*[5.20–6.90] | 239 | 1.81*[1.59–2.06] |

Abbreviation: SMR: standardized mortality ratio; CI: confidence interval

**p*<0.05

^#^See **Supplementary Table 1** for details.

| **Supplementary Table 10** SMR for non–cancer causes of death following bladder cancer diagnosis in 2000–2005 | | | | | | | | |
| --- | --- | --- | --- | --- | --- | --- | --- | --- |
| Non–cancer diseases^#^ | Overall | | <1 year | | 1–5 years | | >5 years | |
|  | Observed | SMR [95% CI] | Observed | SMR [95% CI] | Observed | SMR [95% CI] | Observed | SMR [95% CI] |
| Total | 26325 | 1.88*[1.85–1.90] | 3370 | 29.82*[28.82–30.84] | 8396 | 3.93*[3.85–4.01] | 14559 | 1.24*[1.22–1.26] |
| Infectious diseases | 722 | 2.30*[2.13–2.47] | 142 | 42.04*[35.41–49.55] | 210 | 5.06*[4.40–5.79] | 370 | 1.37*[1.24–1.52] |
| Diabetes Mellitus | 966 | 2.44*[2.29–2.60] | 118 | 33.49*[27.72–40.11] | 318 | 5.00*[4.46–5.58] | 530 | 1.61*[1.48–1.76] |
| Cardiovascular diseases | 12611 | 1.90*[1.87–1.93] | 1828 | 28.58*[27.28–29.92] | 4246 | 3.81*[3.70–3.93] | 6537 | 1.20*[1.17–1.23] |
| Respiratory diseases | 3875 | 2.13*[2.06–2.20] | 496 | 30.98*[28.31–33.83] | 1281 | 4.32*[4.09–4.56] | 2098 | 1.39*[1.33–1.45] |
| Digestive diseases | 230 | 3.67*[3.21–4.18] | 33 | 49.83*[34.30–69.98] | 103 | 6.46*[5.27–7.83] | 94 | 2.04*[1.65–2.50] |
| Other non–cancer diseases | 7921 | 1.65*[1.61–1.68] | 753 | 29.55*[27.48–31.74] | 2238 | 3.69*[3.54–3.85] | 4930 | 1.18*[1.15–1.21] |

Abbreviation: SMR: standardized mortality ratio; CI: confidence interval

**p*<0.05

^#^See **Supplementary Table 1** for details.

| **Supplementary Table 11** SMR for non–cancer causes of death following bladder cancer diagnosis in 2006-2011 | | | | | | | | |
| --- | --- | --- | --- | --- | --- | --- | --- | --- |
| Non–cancer diseases^#^ | Overall | | <1 year | | 1–5 years | | >5 years | |
|  | Observed | SMR [95% CI] | Observed | SMR [95% CI] | Observed | SMR [95% CI] | Observed | SMR [95% CI] |
| Total | 19620 | 2.68*[2.65–2.72] | 3212 | 31.71*[30.62–32.82] | 8382 | 3.99*[3.90–4.07] | 8026 | 1.57*[1.54–1.61] |
| Infectious diseases | 640 | 3.83*[3.54–4.14] | 159 | 43.54*[37.04–50.86] | 262 | 4.74*[4.18–5.35] | 219 | 2.02*[1.77–2.31] |
| Diabetes Mellitus | 683 | 3.39*[3.14–3.66] | 120 | 37.37*[30.98–44.68] | 284 | 4.71*[4.18–5.29] | 279 | 2.03*[1.79–2.28] |
| Cardiovascular diseases | 9014 | 2.60*[2.55–2.66] | 1548 | 29.21*[27.78–30.71] | 3863 | 3.90*[3.77–4.02] | 3603 | 1.49*[1.44–1.54] |
| Respiratory diseases | 2819 | 3.00*[2.89–3.11] | 404 | 34.46*[31.18–37.99] | 1250 | 4.28*[4.05–4.53] | 1165 | 1.83*[1.73–1.94] |
| Digestive diseases | 195 | 4.96*[4.29–5.71] | 35 | 72.20*[50.29–100.41] | 89 | 7.89*[6.34–9.71] | 71 | 2.58*[2.01–3.25] |
| Other non–cancer diseases | 6269 | 2.51*[2.44–2.57] | 946 | 32.36*[30.33–34.49] | 2634 | 3.80*[3.66–3.95] | 2689 | 1.51*[1.45–1.57] |

Abbreviation: SMR: standardized mortality ratio; CI: confidence interval

**p*<0.05

^#^See **Supplementary Table 1** for details.

| **Supplementary Table 12** SMR for non–cancer causes of death following bladder cancer diagnosis in 2012-2017 | | | | | | | | |
| --- | --- | --- | --- | --- | --- | --- | --- | --- |
| Non–cancer diseases^#^ | Overall | | <1 year | | 1–5 years | | >5 years | |
|  | Observed | SMR [95% CI] | Observed | SMR [95% CI] | Observed | SMR [95% CI] | Observed | SMR [95% CI] |
| Total | 9734 | 5.96*[5.84–6.07] | 3261 | 31.81*[30.73–32.92] | 5908 | 4.67*[4.55–4.79] | 565 | 2.11*[1.94–2.29] |
| Infectious diseases | 366 | 9.60*[8.64–10.63] | 171 | 35.90*[30.72–41.71] | 184 | 6.34*[5.45–7.32] | 11 | 2.53*[1.27–4.54] |
| Diabetes Mellitus | 348 | 7.75*[6.95–8.60] | 115 | 41.36*[34.15–49.65] | 223 | 5.92*[5.17–6.75] | 10 | 2.22*[1.07–4.09] |
| Cardiovascular diseases | 4356 | 5.70*[5.53–5.87] | 1471 | 30.29*[28.76–31.88] | 2639 | 4.43*[4.26–4.60] | 246 | 2.04*[1.80–2.31] |
| Respiratory diseases | 1322 | 6.44*[6.10–6.80] | 421 | 33.31*[30.20–36.65] | 824 | 5.01*[4.67–5.36] | 77 | 2.76*[2.17–3.44] |
| Digestive diseases | 126 | 13.51*[11.26–16.09] | 44 | 56.01*[40.70–75.19] | 71 | 10.26*[8.01–12.94] | 11 | 6.81*[3.40–12.18] |
| Other non–cancer diseases | 3216 | 5.62*[5.43–5.82] | 1039 | 31.50*[29.61–33.47] | 1967 | 4.57*[4.37–4.77] | 210 | 1.93*[1.68–2.21] |

Abbreviation: SMR: standardized mortality ratio; CI: confidence interval

**p*<0.05

^#^See **Supplementary Table 1** for details.

| **Supplementary Table 13** SMR for non–cancer causes of death following bladder cancer diagnosis (without surgery) | | | | | | | | |
| --- | --- | --- | --- | --- | --- | --- | --- | --- |
| Non–cancer diseases^#^ | Overall | | <1 year | | 1–5 years | | >5 years | |
|  | Observed | SMR [95% CI] | Observed | SMR [95% CI] | Observed | SMR [95% CI] | Observed | SMR [95% CI] |
| Total | 2710 | 2.89*[2.78–3.00] | 753 | 35.45*[32.97–38.08] | 1043 | 4.14*[3.89–4.39] | 914 | 1.38*[1.29–1.47] |
| Infectious diseases | 91 | 3.23*[2.60–3.96] | 30 | 65.56*[44.23–93.59] | 35 | 6.63*[4.61–9.21] | 26 | 1.16*[0.76–1.70] |
| Diabetes Mellitus | 84 | 3.34*[2.66–4.13] | 27 | 33.81*[22.28–49.19] | 29 | 6.10*[4.08–8.76] | 28 | 1.43*[0.95–2.06] |
| Cardiovascular diseases | 1302 | 2.91*[2.75–3.07] | 374 | 33.16*[29.89–36.70] | 520 | 3.98*[3.65–4.34] | 408 | 1.33*[1.21–1.47] |
| Respiratory diseases | 370 | 2.90*[2.61–3.21] | 93 | 39.05*[31.52–47.84] | 130 | 4.43*[3.70–5.26] | 147 | 1.53*[1.30–1.80] |
| Digestive diseases | 34 | 5.50*[3.81–7.68] | 9 | 51.36*[23.49–97.50] | 13 | 8.72*[4.64–14.91] | 12 | 2.65*[1.37–4.64] |
| Other non–cancer diseases | 829 | 2.74*[2.56–2.93] | 220 | 35.78*[31.21–40.84] | 316 | 3.91*[3.49–4.37] | 293 | 1.36*[1.21–1.52] |

Abbreviation: SMR: standardized mortality ratio; CI: confidence interval

**p*<0.05

^#^See **Supplementary Table 1** for details.

| **Supplementary Table 14** SMR for non–cancer causes of death following bladder cancer diagnosis (undergoing TURBT) | | | | | | | | |
| --- | --- | --- | --- | --- | --- | --- | --- | --- |
| Non–cancer diseases^#^ | Overall | | <1 year | | 1–5 years | | >5 years | |
|  | Observed | SMR [95% CI] | Observed | SMR [95% CI] | Observed | SMR [95% CI] | Observed | SMR [95% CI] |
| Total | 49655 | 2.36*[2.34–2.38] | 8194 | 29.38*[28.74–30.02] | 20578 | 4.04*[3.98–4.09] | 20883 | 1.33*[1.32–1.35] |
| Infectious diseases | 1418 | 3.07*[2.91–3.23] | 328 | 33.16*[29.67–36.95] | 565 | 4.97*[4.57–5.40] | 525 | 1.55*[1.42–1.69] |
| Diabetes Mellitus | 1820 | 3.07*[2.93–3.21] | 302 | 35.91*[31.98–40.20] | 758 | 4.97*[4.63–5.34] | 760 | 1.76*[1.63–1.89] |
| Cardiovascular diseases | 23317 | 2.33*[2.31–2.37] | 4111 | 28.05*[27.20–28.92] | 9800 | 3.91*[3.83–3.99] | 9406 | 1.28*[1.26–1.31] |
| Respiratory diseases | 7231 | 2.66*[2.60–2.72] | 1136 | 31.47*[29.67–33.36] | 3082 | 4.40*[4.25–4.56] | 3013 | 1.52*[1.47–1.58] |
| Digestive diseases | 476 | 4.81*[4.39–5.26] | 86 | 53.23*[42.58–65.74] | 236 | 7.43*[6.51–8.44] | 154 | 2.35*[1.99–2.75] |
| Other non–cancer diseases | 15393 | 2.14*[2.11–2.18] | 2231 | 29.21*[28.01–30.45] | 6137 | 3.85*[3.75–3.94] | 7025 | 1.27*[1.24–1.30] |

Abbreviation: SMR: standardized mortality ratio; CI: confidence interval

**p*<0.05

^#^See **Supplementary Table 1** for details.

| **Supplementary Table 15** SMR for non–cancer causes of death following bladder cancer diagnosis (undergoing PC) | | | | | | | | |
| --- | --- | --- | --- | --- | --- | --- | --- | --- |
| Non–cancer diseases^#^ | Overall | | <1 year | | 1–5 years | | >5 years | |
|  | Observed | SMR [95% CI] | Observed | SMR [95% CI] | Observed | SMR [95% CI] | Observed | SMR [95% CI] |
| Total | 599 | 2.54*[2.34–2.76] | 142 | 30.58*[25.75–36.04] | 211 | 4.61*[4.01–5.28] | 246 | 1.33*[1.17–1.51] |
| Infectious diseases | 15 | 5.51*[3.09–9.09] | 7 | 45.29*[18.21–93.31] | 3 | 3.20*[0.66–9.36] | 5 | 3.07*[1.00–7.16] |
| Diabetes Mellitus | 18 | 3.54*[2.10–5.59] | 4 | 68.60*[18.69–175.65] | 9 | 7.97*[3.65–15.14] | 5 | 1.28*[0.42–2.99] |
| Cardiovascular diseases | 283 | 2.64*[2.34–2.97] | 69 | 26.83*[20.87–33.95] | 99 | 4.88*[3.96–5.94] | 115 | 1.36*[1.13–1.64] |
| Respiratory diseases | 74 | 2.37*[1.86–2.97] | 19 | 35.04*[21.10–54.72] | 23 | 3.94*[2.49–5.90] | 32 | 1.29*[0.88–1.82] |
| Digestive diseases | 3 | 2.01*[0.42–5.88] | – | – | 2 | 14.12*[1.71–50.99] | 1 | 0.74 [0.02–4.13] |
| Other non–cancer diseases | 206 | 2.35*[2.04–2.69] | 43 | 32.65*[23.63–43.98] | 75 | 4.30*[3.39–5.39] | 88 | 1.28*[1.02–1.57] |

Abbreviation: SMR: standardized mortality ratio; CI: confidence interval

**p*<0.05

^#^See **Supplementary Table 1** for details.

| **Supplementary Table 16** SMR for non–cancer causes of death following bladder cancer diagnosis (undergoing RC) | | | | | | | | |
| --- | --- | --- | --- | --- | --- | --- | --- | --- |
| Non–cancer diseases^#^ | Overall | | <1 year | | 1–5 years | | >5 years | |
|  | Observed | SMR [95% CI] | Observed | SMR [95% CI] | Observed | SMR [95% CI] | Observed | SMR [95% CI] |
| Total | 2715 | 3.52*[3.39–3.66] | 754 | 62.80*[58.39–67.44] | 854 | 7.99*[7.46–8.54] | 1107 | 1.70*[1.60–1.80] |
| Infectious diseases | 204 | 7.59*[6.59–8.71] | 107 | 83.02*[68.04–100.32] | 53 | 8.82*[6.60–11.53] | 44 | 2.25*[1.63–3.02] |
| Diabetes Mellitus | 75 | 4.12*[3.24–5.17] | 20 | 80.60*[49.23–124.48] | 29 | 8.86*[5.93–12.72] | 26 | 1.77*[1.16–2.60] |
| Cardiovascular diseases | 1079 | 3.32*[3.13–3.53] | 293 | 57.28*[50.91–64.23] | 329 | 7.55*[6.76–8.41] | 457 | 1.66*[1.51–1.82] |
| Respiratory diseases | 341 | 3.83*[3.44–4.26] | 73 | 53.93*[42.27–67.80] | 120 | 6.79*[5.63–8.12] | 148 | 2.12*[1.79–2.49] |
| Digestive diseases | 38 | 8.14*[5.76–11.17] | 17 | 119.91*[69.85–191.98] | 12 | 15.79*[8.16–27.58] | 9 | 2.39*[1.09–4.54] |
| Other non–cancer diseases | 978 | 3.18*[2.99–3.39] | 244 | 63.21*[55.53–71.66] | 311 | 8.73*[7.78–9.75] | 423 | 1.58*[1.43–1.74] |

Abbreviation: SMR: standardized mortality ratio; CI: confidence interval

**p*<0.05

^#^See **Supplementary Table 1** for details.

| **Supplementary Table 17** SMR for non–cancer causes of death following bladder cancer diagnosis (In situ stage) | | | | | | | | |
| --- | --- | --- | --- | --- | --- | --- | --- | --- |
| Non–cancer diseases^#^ | Overall | | <1 year | | 1–5 years | | >5 years | |
|  | Observed | SMR [95% CI] | Observed | SMR [95% CI] | Observed | SMR [95% CI] | Observed | SMR [95% CI] |
| Total | 30755 | 2.14*[2.12–2.17] | 3586 | 27.11*[26.23–28.02] | 12603 | 3.93*[3.86–4.00] | 14566 | 1.32*[1.30–1.34] |
| Infectious diseases | 821 | 2.54*[2.37–2.72] | 105 | 28.79*[23.55–34.85] | 340 | 4.65*[4.17–5.17] | 376 | 1.53*[1.38–1.69] |
| Diabetes Mellitus | 1103 | 2.82*[2.66–2.99] | 139 | 32.76*[27.54–38.68] | 441 | 5.11*[4.65–5.61] | 523 | 1.74*[1.59–1.89] |
| Cardiovascular diseases | 14351 | 2.11*[2.07–2.14] | 1813 | 25.92*[24.74–27.14] | 5991 | 3.82*[3.73–3.92] | 6547 | 1.27*[1.24–1.30] |
| Respiratory diseases | 4502 | 2.42*[2.35–2.49] | 508 | 29.51*[27.00–32.19] | 1885 | 4.29*[4.10–4.48] | 2109 | 1.50*[1.44–1.57] |
| Digestive diseases | 314 | 4.54*[4.05–5.07] | 46 | 53.47*[39.15–71.33] | 151 | 7.19*[6.09–8.43] | 117 | 2.47*[2.04–2.96] |
| Other non–cancer diseases | 9664 | 1.97*[1.93–2.01] | 975 | 26.83*[25.17–28.57] | 3795 | 3.73*[3.61–3.85] | 4894 | 1.27*[1.24–1.31] |

Abbreviation: SMR: standardized mortality ratio; CI: confidence interval

**p*<0.05

^#^See **Supplementary Table 1** for details.

| **Supplementary Table 18** SMR for non–cancer causes of death following bladder cancer diagnosis (Localized stage) | | | | | | | | |
| --- | --- | --- | --- | --- | --- | --- | --- | --- |
| Non–cancer diseases^#^ | Overall | | <1 year | | 1–5 years | | >5 years | |
|  | Observed | SMR [95% CI] | Observed | SMR [95% CI] | Observed | SMR [95% CI] | Observed | SMR [95% CI] |
| Total | 21685 | 2.71*[2.68–2.75] | 4745 | 30.34*[29.48–31.21] | 9089 | 4.22*[4.13–4.31] | 7851 | 1.38*[1.35–1.41] |
| Infectious diseases | 669 | 3.85*[3.56–4.15] | 224 | 34.30*[29.96–39.10] | 253 | 5.54*[4.88–6.27] | 192 | 1.58*[1.36–1.82] |
| Diabetes Mellitus | 808 | 3.36*[3.13–3.60] | 169 | 36.19*[30.94–42.08] | 356 | 4.97*[4.46–5.51] | 283 | 1.72*[1.53–1.93] |
| Cardiovascular diseases | 10237 | 2.70*[2.65–2.75] | 2371 | 29.02*[27.87–30.22] | 4322 | 4.08*[3.96–4.21] | 3544 | 1.34*[1.29–1.38] |
| Respiratory diseases | 3128 | 3.02*[2.92–3.13] | 656 | 32.79*[30.32–35.39] | 1336 | 4.54*[4.30–4.79] | 1136 | 1.58*[1.49–1.67] |
| Digestive diseases | 207 | 5.14*[4.46–5.89] | 48 | 55.15*[40.67–73.12] | 104 | 8.22*[6.72–9.96] | 55 | 2.05*[1.55–2.67] |
| Other non–cancer diseases | 6636 | 2.44*[2.38–2.50] | 1277 | 29.95*[28.33–31.64] | 2718 | 4.05*[3.90–4.21] | 2641 | 1.32*[1.27–1.37] |

Abbreviation: SMR: standardized mortality ratio; CI: confidence interval

**p*<0.05

^#^See **Supplementary Table 1** for details.

| **Supplementary Table 19** SMR for non–cancer causes of death following bladder cancer diagnosis (Regional stage) | | | | | | | | |
| --- | --- | --- | --- | --- | --- | --- | --- | --- |
| Non–cancer diseases^#^ | Overall | | <1 year | | 1–5 years | | >5 years | |
|  | Observed | SMR [95% CI] | Observed | SMR [95% CI] | Observed | SMR [95% CI] | Observed | SMR [95% CI] |
| Total | 2530 | 4.40*[4.23–4.57] | 1026 | 48.43*[45.51–51.49] | 826 | 6.42*[5.99–6.87] | 678 | 1.59*[1.48–1.72] |
| Infectious diseases | 174 | 9.04*[7.75–10.49] | 99 | 76.89*[62.49–93.61] | 46 | 8.43*[6.17–11.24] | 29 | 2.32*[1.55–3.33] |
| Diabetes Mellitus | 72 | 7.42*[5.81–9.35] | 33 | 90.18*[62.08–126.65] | 26 | 7.29*[4.76–10.68] | 13 | 2.26*[1.20–3.86] |
| Cardiovascular diseases | 1080 | 4.38*[4.12–4.65] | 441 | 42.80*[38.90–46.99] | 363 | 5.44*[4.89–6.03] | 276 | 1.63*[1.44–1.83] |
| Respiratory diseases | 308 | 4.90*[4.37–5.48] | 103 | 42.32*[34.54–51.33] | 117 | 6.95*[5.74–8.32] | 88 | 2.02*[1.62–2.49] |
| Digestive diseases | 21 | 13.08*[8.10–19.99] | 10 | 86.40*[41.43–158.89] | 8 | 15.60*[6.73–30.73] | 3 | 3.07*[0.63–8.97] |
| Other non–cancer diseases | 875 | 3.71*[3.47–3.97] | 340 | 50.93*[45.66–56.64] | 266 | 7.48*[6.61–8.44] | 269 | 1.39*[1.23–1.57] |

Abbreviation: SMR: standardized mortality ratio; CI: confidence interval

**p*<0.05

^#^See **Supplementary Table 1** for details.

| **Supplementary Table 20** SMR for non–cancer causes of death following bladder cancer diagnosis (Distant stage) | | | | | | | | |
| --- | --- | --- | --- | --- | --- | --- | --- | --- |
| Non–cancer diseases^#^ | Overall | | <1 year | | 1–5 years | | >5 years | |
|  | Observed | SMR [95% CI] | Observed | SMR [95% CI] | Observed | SMR [95% CI] | Observed | SMR [95% CI] |
| Total | 709 | 11.52*[10.69–12.40] | 486 | 69.54*[63.5–76.01] | 168 | 9.41*[8.04–10.94] | 55 | 1.50*[1.13–1.95] |
| Infectious diseases | 64 | 16.74*[12.89–21.37] | 44 | 134.49*[97.72–180.55] | 17 | 10.75*[6.26–17.22] | 3 | 1.57*[0.32–4.58] |
| Diabetes Mellitus | 14 | 44.41*[24.28–74.50] | 12 | 50.70*[26.20–88.57] | 2 | 25.44*[3.08–91.91] | – | – |
| Cardiovascular diseases | 313 | 12.65*[11.28–14.13] | 222 | 62.24*[54.32–70.99] | 72 | 8.97*[7.02–11.29] | 19 | 1.44*[0.87–2.26] |
| Respiratory diseases | 78 | 11.33*[8.96–14.14] | 54 | 75.52*[56.74–98.54] | 17 | 8.68*[5.06–13.90] | 7 | 1.66*[0.67–3.43] |
| Digestive diseases | 9 | 55.89*[25.56–106.11] | 8 | 92.69*[40.02–182.63] | – | – | 1 | 13.39*[0.34–74.58] |
| Other non–cancer diseases | 231 | 9.02*[7.89–10.26] | 146 | 70.99*[59.94–83.48] | 60 | 9.66*[7.37–12.43] | 25 | 1.44*[0.93–2.13] |

Abbreviation: SMR: standardized mortality ratio; CI: confidence interval

**p*<0.05

^#^See **Supplementary Table 1** for details.

| **Supplementary Table 21** SMR for non–cancer causes of death following bladder cancer diagnosis (Chemotherapy: No/Unknown) | | | | | | | | |
| --- | --- | --- | --- | --- | --- | --- | --- | --- |
| Non–cancer diseases^#^ | Overall | | <1 year | | 1–5 years | | >5 years | |
|  | Observed | SMR [95% CI] | Observed | SMR [95% CI] | Observed | SMR [95% CI] | Observed | SMR [95% CI] |
| Total | 48977 | 2.34*[2.32–2.36] | 8408 | 30.46*[29.81–31.12] | 19543 | 4.04*[3.98–4.09] | 21026 | 1.33*[1.31–1.35] |
| Infectious diseases | 1451 | 3.08*[2.92–3.24] | 366 | 38.06*[34.26–42.16] | 545 | 5.01*[4.60–5.45] | 540 | 1.53*[1.40–1.66] |
| Diabetes Mellitus | 1791 | 3.07*[2.93–3.22] | 310 | 36.81*[32.83–41.14] | 740 | 5.09*[4.73–5.47] | 741 | 1.72*[1.60–1.85] |
| Cardiovascular diseases | 22936 | 2.32*[2.29–2.35] | 4178 | 28.74*[27.88–29.63] | 9351 | 3.91*[3.83–3.99] | 9407 | 1.28*[1.25–1.30] |
| Respiratory diseases | 7066 | 2.60*[2.54–2.67] | 1138 | 31.90*[30.07–33.81] | 2886 | 4.34*[4.18–4.50] | 3042 | 1.51*[1.46–1.57] |
| Digestive diseases | 471 | 4.58*[4.18–5.02] | 89 | 58.78*[47.20–72.33] | 217 | 7.47*[6.51–8.54] | 165 | 2.29*[1.95–2.66] |
| Other non–cancer diseases | 15262 | 2.12*[2.09–2.16] | 2327 | 30.86*[29.61–32.14] | 5804 | 3.87*[3.77–3.97] | 7131 | 1.27*[1.24–1.30] |

Abbreviation: SMR: standardized mortality ratio; CI: confidence interval

**p*<0.05

^#^See **Supplementary Table 1** for details.

| **Supplementary Table 22** SMR for non–cancer causes of death following bladder cancer diagnosis (Chemotherapy: Yes) | | | | | | | | |
| --- | --- | --- | --- | --- | --- | --- | --- | --- |
| Non–cancer diseases^#^ | Overall | | <1 year | | 1–5 years | | >5 years | |
|  | Observed | SMR [95% CI] | Observed | SMR [95% CI] | Observed | SMR [95% CI] | Observed | SMR [95% CI] |
| Total | 6702 | 3.30*[3.22–3.38] | 1435 | 35.15*[33.36–37.02] | 3143 | 4.74*[4.58–4.91] | 2124 | 1.60*[1.54–1.67] |
| Infectious diseases | 277 | 5.74*[5.08–6.45] | 106 | 48.73*[39.89–58.93] | 111 | 6.47*[5.33–7.80] | 60 | 2.07*[1.58–2.67] |
| Diabetes Mellitus | 206 | 3.51*[3.05–4.02] | 43 | 39.34*[28.47–52.99] | 85 | 5.24*[4.19–6.48] | 78 | 1.89*[1.49–2.35] |
| Cardiovascular diseases | 3045 | 3.16*[3.05–3.27] | 669 | 33.20*[30.73–35.82] | 1397 | 4.55*[4.31–4.79] | 979 | 1.54*[1.44–1.64] |
| Respiratory diseases | 950 | 3.79*[3.55–4.04] | 183 | 38.97*[33.53–45.04] | 469 | 5.34*[4.87–5.85] | 298 | 1.88*[1.67–2.11] |
| Digestive diseases | 80 | 9.41*[7.46–11.71] | 23 | 54.97*[34.84–82.48] | 46 | 8.99*[6.58–11.99] | 11 | 3.71*[1.85–6.63] |
| Other non–cancer diseases | 2144 | 3.07*[2.94–3.20] | 411 | 33.45*[30.29–36.84] | 1035 | 4.51*[4.24–4.80] | 698 | 1.53*[1.41–1.64] |

Abbreviation: SMR: standardized mortality ratio; CI: confidence interval

**p*<0.05

^#^See **Supplementary Table 1** for details.

| **Supplementary Table 23** SMR for non–cancer causes of death following bladder cancer diagnosis (Radiation therapy: No/Unknown) | | | | | | | | |
| --- | --- | --- | --- | --- | --- | --- | --- | --- |
| Non–cancer diseases^#^ | Overall | | <1 year | | 1–5 years | | >5 years | |
|  | Observed | SMR [95% CI] | Observed | SMR [95% CI] | Observed | SMR [95% CI] | Observed | SMR [95% CI] |
| Total | 53334 | 2.38*[2.36–2.40] | 9012 | 31.01*[30.38–31.66] | 21624 | 4.09*[4.04–4.15] | 22698 | 1.35*[1.33–1.37] |
| Infectious diseases | 1629 | 3.28*[3.12–3.44] | 431 | 40.40*[36.67–44.40] | 619 | 5.18*[4.78–5.61] | 579 | 1.58*[1.45–1.71] |
| Diabetes Mellitus | 1919 | 3.07*[2.94–3.21] | 323 | 37.71*[33.71–42.06] | 793 | 5.07*[4.72–5.43] | 803 | 1.75*[1.63–1.87] |
| Cardiovascular diseases | 24859 | 2.35*[2.32–2.37] | 4436 | 29.34*[28.48–30.21] | 10238 | 3.96*[3.88–4.03] | 10185 | 1.30*[1.27–1.32] |
| Respiratory diseases | 7679 | 2.64*[2.59–2.70] | 1196 | 32.18*[30.38–34.06] | 3199 | 4.42*[4.27–4.58] | 3284 | 1.53*[1.48–1.59] |
| Digestive diseases | 532 | 4.91*[4.51–5.35] | 108 | 60.03*[49.24–72.48] | 251 | 7.58*[6.67–8.58] | 173 | 2.36*[2.02–2.74] |
| Other non–cancer diseases | 16716 | 2.18*[2.14–2.21] | 2518 | 31.02*[29.82–32.25] | 6524 | 3.92*[3.82–4.01] | 7674 | 1.29*[1.26–1.32] |

Abbreviation: SMR: standardized mortality ratio; CI: confidence interval

**p*<0.05

^#^See **Supplementary Table 1** for details.

| **Supplementary Table 24** SMR for non–cancer causes of death following bladder cancer diagnosis (Radiation therapy: Yes) | | | | | | | | |
| --- | --- | --- | --- | --- | --- | --- | --- | --- |
| Non–cancer diseases^#^ | Overall | | <1 year | | 1–5 years | | >5 years | |
|  | Observed | SMR [95% CI] | Observed | SMR [95% CI] | Observed | SMR [95% CI] | Observed | SMR [95% CI] |
| Total | 2345 | 4.10*[3.93–4.27] | 831 | 31.66*[29.55–33.89] | 1062 | 4.88*[4.59–5.18] | 452 | 1.38*[1.25–1.51] |
| Infectious diseases | 99 | 4.44*[3.61–5.41] | 41 | 36.50*[26.19–49.51] | 37 | 5.84*[4.11–8.05] | 21 | 1.42*[0.88–2.17] |
| Diabetes Mellitus | 78 | 4.49*[3.55–5.60] | 30 | 31.57*[21.30–45.06] | 32 | 6.39*[4.37–9.02] | 16 | 1.40*[0.80–2.27] |
| Cardiovascular diseases | 1122 | 4.19*[3.95–4.44] | 411 | 28.74*[26.03–31.65] | 510 | 4.57*[4.18–4.99] | 201 | 1.41*[1.23–1.62] |
| Respiratory diseases | 337 | 5.61*[5.03–6.24] | 125 | 38.96*[32.43–46.42] | 156 | 5.38*[4.57–6.29] | 56 | 2.01*[1.52–2.61] |
| Digestive diseases | 19 | 6.33*[3.81–9.89] | 4 | 29.95*[8.16–76.69] | 12 | 11.51*[5.95–20.1] | 3 | 1.64*[0.34–4.81] |
| Other non–cancer diseases | 690 | 3.42*[3.17–3.69] | 220 | 33.71*[29.40–38.47] | 315 | 4.86*[4.34–5.42] | 155 | 1.19*[1.01–1.39] |

Abbreviation: SMR: standardized mortality ratio; CI: confidence interval

**p*<0.05

^#^See **Supplementary Table 1** for details.

| **Supplementary Table 25** SMR for non–cancer causes of death following bladder cancer diagnosis in married patients | | | | | | | | |
| --- | --- | --- | --- | --- | --- | --- | --- | --- |
| Non–cancer diseases^#^ | Overall | | <1 year | | 1–5 years | | >5 years | |
|  | Observed | SMR [95% CI] | Observed | SMR [95% CI] | Observed | SMR [95% CI] | Observed | SMR [95% CI] |
| Total | 32992 | 2.29*[2.27–2.32] | 5163 | 31.77*[30.91–32.65] | 13057 | 4.17*[4.09–4.24] | 14772 | 1.33*[1.31–1.35] |
| Infectious diseases | 959 | 3.04*[2.85–3.24] | 230 | 37.37*[32.69–42.52] | 365 | 5.01*[4.51–5.55] | 364 | 1.54*[1.38–1.71] |
| Diabetes Mellitus | 1237 | 2.89*[2.73–3.05] | 192 | 40.52*[34.99–46.68] | 485 | 5.16*[4.71–5.64] | 560 | 1.70*[1.56–1.84] |
| Cardiovascular diseases | 15465 | 2.27*[2.24–2.31] | 2593 | 30.26*[29.10–31.44] | 6257 | 4.06*[3.96–4.16] | 6615 | 1.28*[1.25–1.31] |
| Respiratory diseases | 4528 | 2.51*[2.43–2.58] | 660 | 33.40*[30.90–36.05] | 1782 | 4.43*[4.23–4.65] | 2086 | 1.51*[1.44–1.57] |
| Digestive diseases | 306 | 4.76*[4.24–5.33] | 57 | 58.70*[44.46–76.06] | 142 | 7.61*[6.41–8.96] | 107 | 2.40*[1.97–2.90] |
| Other non–cancer diseases | 10497 | 2.11*[2.07–2.15] | 1431 | 31.66*[30.04–33.35] | 4026 | 4.00*[3.87–4.12] | 5040 | 1.29*[1.25–1.32] |

Abbreviation: SMR: standardized mortality ratio; CI: confidence interval

**p*<0.05

^#^See **Supplementary Table 1** for details.

| **Supplementary Table 26** SMR for non–cancer causes of death following bladder cancer diagnosis in separated patients | | | | | | | | |
| --- | --- | --- | --- | --- | --- | --- | --- | --- |
| Non–cancer diseases | Overall | | <1 year | | 1–5 years | | >5 years | |
|  | Observed | SMR [95% CI] | Observed | SMR [95% CI] | Observed | SMR [95% CI] | Observed | SMR [95% CI] |
| Total | 323 | 3.33*[2.98–3.71] | 47 | 57.94*[42.57–77.05] | 144 | 6.02*[5.08–7.09] | 132 | 1.83*[1.53–2.17] |
| Infectious diseases | 17 | 7.16*[4.17–11.46] | 6 | 68.53*[25.15–149.16] | 4 | 34.49*[9.40–88.32] | 7 | 3.22*[1.3–6.64] |
| Diabetes Mellitus | 10 | 3.13*[1.50–5.76] | 3 | 74.76*[15.42–218.48] | 3 | 3.00*[0.62–8.75] | 4 | 1.86*[0.51–4.76] |
| Cardiovascular diseases | 128 | 3.73*[3.11–4.43] | 18 | 52.28*[30.98–82.62] | 66 | 5.50*[4.25–6.99] | 44 | 2.00*[1.45–2.69] |
| Respiratory diseases | 57 | 3.08*[2.33–3.99] | 4 | 165.13*[44.99–422.79] | 28 | 6.54*[4.35–9.45] | 25 | 1.76*[1.14–2.6] |
| Digestive diseases | 8 | 21.73*[9.38–42.81] | 3 | – | 4 | 11.92*[3.25–30.52] | 1 | 31.65[0.80–176.35] |
| Other non–cancer diseases | 103 | 2.70*[2.20–3.27] | 13 | 41.41*[22.05–70.82] | 39 | 6.31*[4.49–8.63] | 51 | 1.61*[1.2–2.11] |

Abbreviation: SMR: standardized mortality ratio; CI: confidence interval

**p*<0.05

^#^See **Supplementary Table 1** for details.

| **Supplementary Table 27** SMR for non–cancer causes of death following bladder cancer diagnosis in divorced patients | | | | | | | | |
| --- | --- | --- | --- | --- | --- | --- | --- | --- |
| Non–cancer diseases^#^ | Overall | | <1 year | | 1–5 years | | >5 years | |
|  | Observed | SMR [95% CI] | Observed | SMR [95% CI] | Observed | SMR [95% CI] | Observed | SMR [95% CI] |
| Total | 3924 | 3.66*[3.55–3.78] | 733 | 51.92*[48.22–55.81] | 1619 | 6.40*[6.10–6.72] | 1572 | 1.96*[1.86–2.05] |
| Infectious diseases | 132 | 5.93*[4.96–7.04] | 39 | 70.49*[50.13–96.37] | 56 | 9.21*[6.96–11.96] | 37 | 2.37*[1.67–3.27] |
| Diabetes Mellitus | 160 | 5.13*[4.36–5.99] | 25 | 40.56*[26.25–59.88] | 66 | 6.98*[5.40–8.88] | 69 | 3.27*[2.54–4.13] |
| Cardiovascular diseases | 1720 | 3.65*[3.48–3.83] | 343 | 48.20*[43.23–53.58] | 722 | 5.88*[5.46–6.32] | 655 | 1.92*[1.77–2.07] |
| Respiratory diseases | 671 | 4.05*[3.75–4.37] | 121 | 66.57*[55.24–79.54] | 284 | 6.99*[6.20–7.86] | 266 | 2.16*[1.91–2.43] |
| Digestive diseases | 68 | 6.27*[4.87–7.96] | 12 | 121.50*[62.78–212.23] | 31 | 11.75*[7.99–16.68] | 25 | 3.09*[2.00–4.56] |
| Other non–cancer diseases | 1173 | 3.17*[2.99–3.36] | 193 | 49.27*[42.57–56.74] | 460 | 6.46*[5.89–7.08] | 520 | 1.77*[1.62–1.92] |

Abbreviation: SMR: standardized mortality ratio; CI: confidence interval

**p*<0.05

^#^See **Supplementary Table 1** for details.

| **Supplementary Table 28** SMR for non–cancer causes of death following bladder cancer diagnosis in widowed patients | | | | | | | | |
| --- | --- | --- | --- | --- | --- | --- | --- | --- |
| Non–cancer diseases^#^ | Overall | | <1 year | | 1–5 years | | >5 years | |
|  | Observed | SMR [95% CI] | Observed | SMR [95% CI] | Observed | SMR [95% CI] | Observed | SMR [95% CI] |
| Total | 13497 | 2.26*[2.22–2.30] | 2866 | 24.70*[23.80–25.62] | 5885 | 3.38*[3.29–3.47] | 4746 | 1.15*[1.12–1.19] |
| Infectious diseases | 402 | 2.89*[2.61–3.19] | 128 | 31.53*[26.30–37.48] | 145 | 3.88*[3.28–4.57] | 129 | 1.32*[1.10–1.57] |
| Diabetes Mellitus | 396 | 2.79*[2.53–3.08] | 89 | 28.63*[22.99–35.23] | 179 | 4.04*[3.47–4.68] | 128 | 1.36*[1.13–1.61] |
| Cardiovascular diseases | 6442 | 2.26*[2.21–2.32] | 1422 | 23.74*[22.52–25.00] | 2829 | 3.31*[3.19–3.44] | 2191 | 1.13*[1.08–1.18] |
| Respiratory diseases | 2007 | 2.59*[2.48–2.71] | 406 | 24.89*[22.53–27.43] | 938 | 3.71*[3.48–3.96] | 663 | 1.31*[1.21–1.42] |
| Digestive diseases | 87 | 3.67*[2.94–4.52] | 20 | 34.64*[21.16–53.50] | 50 | 5.37*[3.99–7.08] | 17 | 1.23*[0.72–1.97] |
| Other non–cancer diseases | 4163 | 2.04*[1.97–2.10] | 801 | 24.97*[23.27–26.76] | 1744 | 3.21*[3.06–3.36] | 1618 | 1.10*[1.05–1.16] |

Abbreviation: SMR: standardized mortality ratio; CI: confidence interval

**p*<0.05

^#^See **Supplementary Table 1** for details.

| **Supplementary Table 29** SMR for non–cancer causes of death following bladder cancer diagnosis in unmarried patients | | | | | | | | |
| --- | --- | --- | --- | --- | --- | --- | --- | --- |
| Non–cancer diseases^#^ | Overall | | <1 year | | 1–5 years | | >5 years | |
|  | Observed | SMR [95% CI] | Observed | SMR [95% CI] | Observed | SMR [95% CI] | Observed | SMR [95% CI] |
| Total | 4943 | 3.39*[3.29–3.48] | 1034 | 44.32*[41.66–47.11] | 1981 | 5.64*[5.39–5.89] | 1928 | 1.78*[1.70–1.86] |
| Infectious diseases | 218 | 5.41*[4.71–6.17] | 69 | 73.70*[57.34–93.27] | 86 | 9.11*[7.28–11.25] | 63 | 2.10*[1.62–2.69] |
| Diabetes Mellitus | 194 | 5.22*[4.51–6.00] | 44 | 43.51*[31.61–58.41] | 92 | 7.20*[5.80–8.83] | 58 | 2.48*[1.88–3.20] |
| Cardiovascular diseases | 2226 | 3.14*[3.01–3.27] | 471 | 37.85*[34.51–41.43] | 874 | 5.11*[4.77–5.46] | 881 | 1.68*[1.57–1.79] |
| Respiratory diseases | 753 | 3.80*[3.53–4.08] | 130 | 52.85*[44.15–62.75] | 323 | 6.03*[5.39–6.72] | 300 | 2.11*[1.88–2.36] |
| Digestive diseases | 82 | 6.79*[5.40–8.42] | 20 | 70.33*[42.96–108.62] | 36 | 11.22*[7.86–15.54] | 26 | 3.03*[1.98–4.43] |
| Other non–cancer diseases | 1470 | 3.18*[3.02–3.35] | 300 | 48.42*[43.10–54.22] | 570 | 5.62*[5.17–6.10] | 600 | 1.69*[1.56–1.83] |

Abbreviation: SMR: standardized mortality ratio; CI: confidence interval

**p*<0.05

^#^See **Supplementary Table 1** for details.

| **Supplementary Table 30** SMR for non–cancer causes of death following bladder cancer diagnosis ( histologic type: Tcc) | | | | | | | | |
| --- | --- | --- | --- | --- | --- | --- | --- | --- |
| Non–cancer diseases^#^ | Overall | | <1 year | | 1–5 years | | >5 years | |
|  | Observed | SMR [95% CI] | Observed | SMR [95% CI] | Observed | SMR [95% CI] | Observed | SMR [95% CI] |
| Total | 53672 | 2.40*[2.38–2.42] | 9144 | 30.36*[29.74–30.99] | 21994 | 4.10*[4.05–4.16] | 22534 | 1.35*[1.33–1.36] |
| Infectious diseases | 1618 | 3.22*[3.07–3.38] | 420 | 38.16*[34.60–42.00] | 62 | 5.11*[4.72–5.53] | 574 | 1.56*[1.43–1.69] |
| Diabetes Mellitus | 1937 | 3.09*[2.95–3.23] | 332 | 36.94*[33.07–41.14] | 804 | 5.11*[4.76–5.47] | 801 | 1.74*[1.62–1.86] |
| Cardiovascular diseases | 25120 | 2.37*[2.34–2.40] | 4547 | 28.84*[28.01–29.69] | 10450 | 3.97*[3.89–4.05] | 10123 | 1.30*[1.27–1.32] |
| Respiratory diseases | 7758 | 2.67*[2.62–2.73] | 1240 | 31.85*[30.10–33.67] | 3262 | 4.46*[4.31–4.61] | 3256 | 1.53*[1.48–1.58] |
| Digestive diseases | 525 | 4.84*[4.43–5.27] | 101 | 57.54*[46.86–69.91] | 255 | 7.59*[6.69–8.58] | 169 | 2.31*[1.98–2.69] |
| Other non–cancer diseases | 16749 | 2.18*[2.15–2.22] | 2519 | 30.23*[29.06–31.43] | 6611 | 3.92*[3.83–4.02] | 7619 | 1.29*[1.26–1.32] |

Abbreviation: SMR: standardized mortality ratio; CI: confidence interval

**p*<0.05

^#^See **Supplementary Table 1** for details.

| **Supplementary Table 31** SMR for non–cancer causes of death following bladder cancer diagnosis ( histologic type: Scc) | | | | | | | | |
| --- | --- | --- | --- | --- | --- | --- | --- | --- |
| Non–cancer diseases^#^ | Overall | | <1 year | | 1–5 years | | >5 years | |
|  | Observed | SMR [95% CI] | Observed | SMR [95% CI] | Observed | SMR [95% CI] | Observed | SMR [95% CI] |
| Total | 804 | 3.11*[2.90–3.33] | 272 | 47.98*[42.44–54.03] | 247 | 4.63*[4.07–5.24] | 285 | 1.43*[1.27–1.60] |
| Infectious diseases | 53 | 5.48*[4.11–7.17] | 25 | 68.48*[44.32–101.09] | 15 | 5.63*[3.15–9.28] | 13 | 1.96*[1.04–3.35] |
| Diabetes Mellitus | 24 | 3.98*[2.55–5.92] | 8 | 192.17*[82.96–378.64] | 7 | 6.03*[2.42–12.42] | 9 | 1.87*[0.85–3.54] |
| Cardiovascular diseases | 327 | 2.79*[2.49–3.11] | 109 | 39.78*[32.66–47.98] | 106 | 4.31*[3.53–5.22] | 112 | 1.24*[1.03–1.50] |
| Respiratory diseases | 112 | 4.55*[3.75–5.48] | 37 | 59.84*[42.14–82.49] | 34 | 4.45*[3.08–6.22] | 41 | 2.51*[1.80–3.40] |
| Digestive diseases | 5 | 4.61*[1.5–10.75] | 1 | – | 2 | 7.99*[0.97–28.86] | 2 | 2.40*[0.29–8.66] |
| Other non–cancer diseases | 283 | 2.83*[2.51–3.18] | 92 | 48.34*[38.97–59.29] | 83 | 4.87*[3.88–6.03] | 108 | 1.33*[1.09–1.61] |

Abbreviation: SMR: standardized mortality ratio; CI: confidence interval

**p*<0.05

^#^See **Supplementary Table 1** for details.

| **Supplementary Table 32** SMR for non–cancer causes of death following bladder cancer diagnosis ( histologic type: Nec) | | | | | | | | |
| --- | --- | --- | --- | --- | --- | --- | --- | --- |
| Non–cancer diseases^#^ | Overall | | <1 year | | 1–5 years | | >5 years | |
|  | Observed | SMR [95% CI] | Observed | SMR [95% CI] | Observed | SMR [95% CI] | Observed | SMR [95% CI] |
| Total | 203 | 4.68*[4.06–5.37] | 96 | 53.45*[43.29–65.27] | 66 | 5.91*[4.57–7.52] | 41 | 1.35*[0.97–1.83] |
| Infectious diseases | 14 | 42.02*[22.97–70.51] | 9 | 84.79*[38.77–160.95] | 4 | 32.87*[8.96–84.17] | 1 | 9.49*[0.24–52.9] |
| Diabetes Mellitus | 4 | 5.88*[1.60–15.07] | 2 | 20.99*[2.54–75.84] | 1 | 4.24*[0.11–23.60] | 1 | 2.87*[0.07–15.99] |
| Cardiovascular diseases | 86 | 4.15*[3.32–5.13] | 39 | 40.53*[28.82–55.40] | 28 | 5.50*[3.65–7.94] | 19 | 1.30*[0.78–2.02] |
| Respiratory diseases | 25 | 7.04*[4.56–10.39] | 9 | 90.88*[41.56–172.52] | 11 | 5.36*[2.68–9.60] | 5 | 3.57*[1.16–8.33] |
| Digestive diseases | 7 | 9.89*[3.98–20.37] | 5 | 45.79*[14.87–106.85] | – | – | 2 | 3.34*[0.40–12.07] |
| Other non–cancer diseases | 67 | 3.86*[2.99–4.90] | 32 | 75.45*[51.61–106.51] | 22 | 6.01*[3.76–9.09] | 13 | 0.98 [0.52–1.67] |

Abbreviation: SMR: standardized mortality ratio; CI: confidence interval

**p*<0.05

^#^See **Supplementary Table 1** for details.

| **Supplementary Table 33** SMR for non–cancer causes of death following bladder cancer diagnosis ( histologic type: Ac) | | | | | | | | |
| --- | --- | --- | --- | --- | --- | --- | --- | --- |
| Non–cancer diseases^#^ | Overall | | <1 year | | 1–5 years | | >5 years | |
|  | Observed | SMR [95% CI] | Observed | SMR [95% CI] | Observed | SMR [95% CI] | Observed | SMR [95% CI] |
| Total | 352 | 3.66*[3.29–4.06] | 105 | 39.05*[31.94–47.27] | 144 | 6.14*[5.18–7.23] | 103 | 1.47*[1.20–1.78] |
| Infectious diseases | 14 | 7.59*[4.15–12.74] | 8 | 40.50*[17.49–79.81] | 5 | 13.58*[4.41–31.7] | 1 | 0.78 [0.02–4.36] |
| Diabetes Mellitus | 11 | 6.05*[3.02–10.82] | 3 | 111.26*[22.94–325.15] | 5 | 8.10*[2.63–18.89] | 3 | 2.55*[0.53–7.46] |
| Cardiovascular diseases | 166 | 3.56*[3.04–4.15] | 51 | 33.98*[25.30–44.68] | 64 | 5.21*[4.01–6.66] | 51 | 1.55*[1.16–2.04] |
| Respiratory diseases | 40 | 3.42*[2.44–4.66] | 7 | 25.58*[10.28–52.71] | 20 | 5.62*[3.43–8.69] | 13 | 1.650[0.88–2.82] |
| Digestive diseases | 7 | 12.38*[4.98–25.50] | 3 | 87.41*[18.03–255.46] | 2 | 16.09*[1.95–58.13] | 2 | 4.91*[0.60–17.75] |
| Other non–cancer diseases | 114 | 3.38*[2.79–4.06] | 33 | 50.31*[34.63–70.66] | 48 | 7.38*[5.44–9.78] | 33 | 1.24*[0.86–1.75] |

Abbreviation: SMR: standardized mortality ratio; CI: confidence interval

**p*<0.05

^#^See **Supplementary Table 1** for details.

| **Supplementary Table 34** SMR for non–cancer causes of death following bladder cancer diagnosis ( histologic type: Oet) | | | | | | | | |
| --- | --- | --- | --- | --- | --- | --- | --- | --- |
| Non–cancer diseases^#^ | Overall | | <1 year | | 1–5 years | | >5 years | |
|  | Observed | SMR [95% CI] | Observed | SMR [95% CI] | Observed | SMR [95% CI] | Observed | SMR [95% CI] |
| Total | 613 | 3.31*[3.05–3.58] | 211 | 40.49*[35.21–46.33] | 223 | 4.03*[3.52–4.59] | 179 | 1.44*[1.23–1.66] |
| Infectious diseases | 29 | 4.68*[3.13–6.72] | 10 | 74.51*[35.73–137.03] | 8 | 13.16*[5.68–25.93] | 11 | 2.02*[1.01–3.61] |
| Diabetes Mellitus | 21 | 3.08*[1.91–4.71] | 9 | 23.09*[10.56–43.82] | 7 | 3.28*[1.32–6.75] | 5 | 1.17*[0.38–2.72] |
| Cardiovascular diseases | 282 | 3.21*[2.85–3.61] | 99 | 36.58*[29.73–44.54] | 102 | 3.95*[3.22–4.80] | 81 | 1.37*[1.08–1.70] |
| Respiratory diseases | 81 | 3.52*[2.79–4.37] | 28 | 62.30*[41.40–90.04] | 28 | 3.52*[2.34–5.09] | 25 | 1.71*[1.11–2.52] |
| Digestive diseases | 7 | 16.50*[6.63–33.99] | 2 | 61.61*[7.46–222.54] | 4 | 20.85*[5.68–53.38] | 1 | 5.00*[0.13–27.86] |
| Other non–cancer diseases | 193 | 3.17*[2.74–3.65] | 63 | 42.02*[32.29–53.76] | 74 | 3.96*[3.11–4.97] | 56 | 1.37*[1.04–1.78] |

Abbreviation: SMR: standardized mortality ratio; CI: confidence interval

**p*<0.05

^#^See **Supplementary Table 1** for details.
